# Dynamic phenotyping of radioiodine-refractory differentiated thyroid cancer: thyroglobulin trajectories, dual-modal imaging, and genotype-defined treatment resistance

**DOI:** 10.3389/fendo.2026.1891291

**Published:** 2026-08-10

**Authors:** Gongxun Tan, Yue Zhang, Zhenwei Mao, Chuan Zhang, Jingjing Fu, Feng Wang, Canhong Zhu, Jin Zhou, Bingquan Jin, Ying Xu, Rong Qin, Xun Shao, Lele Zhang, Shaohua Li, Qian Gu

**Affiliations:** 1 Department of Geriatrics, Affiliated People’s Hospital of Jiangsu University, Zhenjiang, Jiangsu, China; 2 Department of Nuclear Medicine, Shuyang Hospital of Chinese Traditional Medicine, Shuyang, Jiangsu, China; 3 Department of Laboratory, Affiliated Fourth People’s Hospital of Jiangsu University, Zhenjiang, Jiangsu, China; 4 Department of Nuclear Medicine, Nanjing First Hospital, Nanjing Medical University, Nanjing, Jiangsu, China; 5 Nanjing D.A. Medical Laboratory, Nanjing, Jiangsu, China; 6 Department of Oncology, Affiliated People’s Hospital of Jiangsu University, Zhenjiang, Jiangsu, China

**Keywords:** ^18^F-FDG PET/CT, *BRAFV600E*, dual-modal imaging, radioiodine-refractory differentiated thyroid cancer, stimulated thyroglobulin, TERT promoter mutation, tyrosine kinase inhibitor resistance

## Abstract

**Background:**

Radioiodine-refractory differentiated thyroid cancer (RAIR-DTC) is a clinically aggressive disease state with limited treatment options. While individual biomarkers, imaging modalities, and genomic drivers have each been studied in isolation, the dynamic relationships among longitudinal thyroglobulin kinetics, evolving dual-modal imaging phenotypes, and underlying genotypes, together with their consequences for targeted therapy response, remain insufficiently characterized.

**Methods:**

We retrospectively analyzed 424 patients with intermediate- to high-risk differentiated thyroid cancer. Longitudinal serial stimulated thyroglobulin (sTg) measurements, paired ¹³¹I whole-body scan and ¹^8^F-FDG PET/CT, and next-generation sequencing of postoperative tumor tissue were integrated. Imaging-defined phenotypes (¹³¹I-avid, ¹^8^F-FDG-avid, or dual-avid) were stratified across sTg levels and correlated with metastatic distribution, genotype, and response to first-line tyrosine kinase inhibitors (TKIs).

**Results:**

Sixty-four patients (15.1%) progressed to RAIR-DTC. Median sTg rose from 18.7 ng/mL at initial radioiodine therapy to 302.7 ng/mL at RAIR-DTC diagnosis; pre-radioiodine sTg discriminated RAIR-DTC progression with an area under the curve of 0.911 (95% CI 0.856–0.967; optimal threshold 10.77 ng/mL). With increasing sTg, imaging phenotypes evolved non-binarily, shifting from mixed ¹³¹I/¹^8^F-FDG-avid lesions toward predominantly ¹^8^F-FDG-avid disease; metastatic co-occurrence network analysis identified cervical lymph nodes and bilateral lungs as central dissemination hubs. *TERT* promoter (*TERTp*) mutations were significantly enriched in RAIR-DTC (43.8% vs 5.3% in non-RAIR-DTC; *p* < 0.001) and clustered with *BRAFV600E* within a tightly connected oncogenic network; patients harboring *TERTp* mutations had markedly shorter progression-free survival (median 18.2 vs 47.6 months; *p* < 0.0001). Lesions in patients with *BRAFV600E*/*TERTp* co-mutation exhibited significantly higher metabolic activity (maximum standardized uptake value; *p* < 0.0001) and earlier radiographic progression on first-line TKIs (68.4% vs 23.5% within six months; *p* = 0.001).

**Conclusion:**

Coupling longitudinal sTg kinetics with evolving dual-modal imaging phenotypes delineates genotype-associated disease courses in RAIR-DTC, in which *TERTp* mutation, particularly when co-occurring with *BRAFV600E*, characterizes a metabolically aggressive, TKI-resistant subset. These findings support a dynamic phenotyping approach for refining surveillance and informing genotype-guided treatment decisions in RAIR-DTC.

## Introduction

Differentiated thyroid carcinoma (DTC) is the most common endocrine malignancy (1). Standard management, including surgery, adjuvant radioactive iodine (RAI) therapy, and thyroid-stimulating hormone (TSH) suppression, yields a favorable prognosis for most patients. However, approximately 5–15% of patients eventually progress to radioiodine-refractory DTC (RAIR-DTC). This transition marks a critical turning point, as the loss of iodine avidity renders RAI treatment ineffective and leads to a dramatic decline in clinical outcomes, with 10-year survival rates falling from greater than 90% to less than 10%. Consequently, RAIR-DTC accounts for the vast majority of thyroid cancer-related deaths and represents one of the most pressing clinical challenges in DTC management (2).

RAIR pathogenesis is a multistep process driven by molecular dedifferentiation, with *BRAFV600E* and TERT promoter (*TERTp*) mutations as the principal genomic drivers. *BRAFV600E* dysregulates the MAPK pathway and downregulates the sodium-iodide symporter (NIS), whereas *TERTp* mutations are strongly associated with tumor aggressiveness. Co-occurring alterations, particularly *BRAFV600E*/*TERTp* co-mutations, exhibit a synergistic effect, promoting a more aggressive phenotype and a higher risk of RAI resistance (3). Current research is often constrained by a “single-gene” perspective, failing to capture the complex co-mutational landscape that defines RAIR-DTC (4). Furthermore, a critical disconnect persists between genomic alterations and their corresponding clinical phenotypes. Early prediction strategies largely rely on static clinicopathological variables or isolated biomarkers (5), without dynamically integrating genomic features with longitudinal clinical trajectories, such as serial serum stimulated thyroglobulin (sTg) kinetics, evolving imaging phenotypes (¹^8^F-fluorodeoxyglucose positron emission tomography-computed tomography [¹^8^F-FDG PET/CT] and ¹³¹I whole-body scan [¹³¹I-WBS]), metastatic patterns, and treatment history. This disconnection hinders mechanistic understanding of dedifferentiation and limits the ability to identify subgroups at risk of targeted therapy resistance.

To address these gaps, we conducted a retrospective cohort study of 424 patients with intermediate- to high-risk DTC. Our objectives were threefold: (i) to characterize the dynamic evolution of sTg kinetics and dual-modal imaging phenotypes during progression from intermediate- to high-risk DTC to RAIR-DTC, including metastatic dissemination patterns; (ii) to define the genomic landscape underlying these phenotypes, with particular focus on *TERTp* mutation and *BRAFV600E*/*TERTp* co-mutation; and (iii) to examine how these genotype-defined phenotypes translate into response to first-line tyrosine kinase inhibitor (TKI) therapy. By integrating these three dimensions in a single cohort, this study delineates the disease course from RAI-sensitive DTC to TKI-resistant RAIR-DTC and provides a phenotype-genotype framework to inform surveillance and treatment decisions.

## Materials and methods

### Patient selection and inclusion criteria

The study protocol was approved by the Ethics Committee of Nanjing First Hospital Affiliated with Nanjing Medical University. Written informed consent was waived due to the retrospective nature of the study. We screened 2,224 patients with thyroid carcinoma who underwent total or near-total thyroidectomy between 2014 and 2025 (follow-up until December 31, 2025). Recurrence risk was stratified according to the 2025 American Thyroid Association (ATA) Management Guidelines for Adult Patients with Differentiated Thyroid Carcinoma (6). Risk was categorized as low, intermediate-low, intermediate-high, or high based on clinicopathological features, including tumor size, extrathyroidal extension (ETE), and lymph node metastasis (LNM) status (**Supplementary Table 1**). All patients received TSH suppression therapy, and those with intermediate-high or high recurrence risk additionally underwent RAI. Exclusion criteria included low or intermediate-low recurrence risk, incomplete clinical records, missing RAI-related data, or insufficient follow-up information.

A total of 424 patients with intermediate- to high-risk DTC were included in the final analysis (**Supplementary Table 2**). Retrospectively collected data included age, sex, gene mutations, serology, histology, maximum tumor diameter, multifocality, LNM, distant metastasis, ¹^8^F-FDG PET/CT, ¹³¹I-WBS, follow-up duration, and time from initial RAI to RAIR-DTC diagnosis.

### Diagnostic criteria for RAIR-DTC and targeted therapy resistance

Diagnosis was based on the 2015 ATA Guidelines (7) and the Chinese Society of Clinical Oncology (CSCO) guidelines for persistent/recurrent and metastatic differentiated thyroid cancer (8). Patients were categorized into non-RAIR-DTC (n = 360) and RAIR-DTC (n = 64). For this study, RAIR-DTC was operationally defined when a patient fulfilled at least one of five non-mutually exclusive criteria: (1) absence of radioiodine uptake in known metastatic or structurally persistent disease from the first post-therapeutic ¹³¹I evaluation; (2) loss of previously documented radioiodine avidity in one or more lesions during follow-up; (3) spatial heterogeneity of iodine avidity, with radioiodine uptake retained in some lesions but absent in other structurally evident lesions; (4) structural disease progression, defined by enlargement of known lesions or appearance of new lesions on cross-sectional or metabolic imaging, despite demonstrable radioiodine uptake and adequate RAI treatment; or (5) persistent/progressive disease after a cumulative RAI activity ≥600 mCi (22.2 GBq). The criteria were not mutually exclusive, and classification was based on the complete longitudinal clinical, biochemical, and imaging record rather than on sTg elevation alone.

Patients were evaluated using neck ultrasound, chest CT, ¹^8^F-FDG PET/CT, bone scintigraphy, ¹³¹I-WBS, serial changes in sTg and thyroglobulin antibody (TgAb) levels from the initial RAI to RAIR-DTC diagnosis, and assessment of lymph-node and distant metastases. Stimulated serologic assessments were performed after thyroid hormone withdrawal with TSH >30 mIU/L and without known exogenous iodine interference. Post-therapeutic ¹³¹I-WBS was acquired at the protocol-defined time point 4 days after RAI administration. In contrast, because of the retrospective real-world design, ¹^8^F-FDG PET/CT and other cross-sectional imaging were clinically indicated and were not obtained at fixed calendar intervals for every patient. The longitudinal RAIR-DTC classification therefore used all available serial examinations and required concordant structural/imaging evidence for progression when Criterion 4 was applied. For dual-modal phenotyping, a ¹³¹I-WBS(+)/FDG(+) (“dual-avid” or dual-positive) phenotype was defined at the patient-assessment level as the presence of at least one pathological radioiodine-avid lesion and at least one pathological FDG-avid lesion during the corresponding dual-modal evaluation; the two signals were not required to arise from the same anatomical lesion. Thus, spatially discordant lesions could contribute to the dual-positive phenotype. Detailed case-by-case diagnostic bases for the 64 RAIR-DTC patients are provided in **Supplementary Note 1** and **Supplementary Tables 3** and **4**.

Among patients with RAIR-DTC receiving first-line targeted therapy, ¹^8^F-FDG PET/CT was used to evaluate treatment response. Resistance was defined as disease progression following an initial response to targeted therapy.

### Postoperative RAI and serologic testing

All patients in this study underwent thyroid hormone withdrawal (levothyroxine sodium) for 3–4 weeks combined with a low-iodine diet until TSH >30 mU/L. An appropriate RAI dose (3.7–7.4 GBq) was administered based on clinical indications. Serum TgAb, sTg, and TSH were measured using electrochemiluminescence immunoassay (Roche E170, Switzerland) or chemiluminescent immunoassay (ADVIA Centaur, Bayer, Germany), according to the laboratory platform available during the study period. The reported analytical measurement range for sTg was 0.04–1000.00 ng/mL (reference range, 1.40–78.00 ng/mL), with a manufacturer-reported analytical sensitivity of 0.04 ng/mL. Values below or above the measurement range were recorded as 0.04 ng/mL or 1000.00 ng/mL, respectively, for statistical analysis. TgAb was assessed concurrently; the reference range was 0–115 kU/L, and values <115 kU/L were considered negative. To minimize interference from anti-thyroglobulin antibodies, six patients with elevated TgAb were excluded from the sTg-based analyses. The TSH reference range was 0.27–4.20 mU/L. All patients received levothyroxine TSH suppression therapy during and after RAI treatment, with a target TSH level <0.1 mU/L. The sTg levels at the first RAI and at RAIR-DTC diagnosis were used as dynamic serological indicators. Because two immunoassay platforms were used over the retrospective study period and no formal cross-platform calibration study was performed, residual inter-assay variability cannot be excluded; this issue was considered when interpreting absolute sTg thresholds.

### ¹³¹I whole-body scan and SPECT/CT

#### Instrumentation and reagents

Imaging was performed using a Siemens Symbia T6 SPECT/CT scanner (Siemens Healthineers, Germany) equipped with a high-energy general-purpose parallel-hole collimator. The imaging agent was an oral solution of ¹³¹I with a radionuclidic purity greater than 99% (Chengdu Zhonghe Gaotong Isotope Co., Ltd., China).

#### Imaging protocol

Patients underwent ¹³¹I-WBS and regional SPECT/CT fusion imaging 4 days after ¹³¹I therapy. For ¹³¹I-WBS, patients were positioned supine and scanning was performed at a speed of 18 cm/min using a 256 × 1024 matrix; the energy peak was set at 364 keV with a 20% window, and anterior and posterior projection images were acquired simultaneously. Based on the WBS findings, SPECT/CT tomographic imaging was then performed for regions of interest such as the neck, thorax, or any suspected foci of abnormal radiotracer uptake. SPECT acquisition used a high-energy general-purpose parallel-hole collimator with the energy window centered at 364 keV ± 10%, a 128 × 128 matrix, an acquisition time of approximately 30 seconds per frame, and automatic body contour tracking. CT acquisition was performed with the integrated low-dose spiral CT (tube voltage 120 kV, tube current 80 mA, slice thickness 3.75 mm) and used for anatomical localization and attenuation correction.

#### Image processing

Image reconstruction and fusion were performed on the manufacturer’s dedicated workstation. SPECT images were iteratively reconstructed using the ordered-subsets expectation maximization (OSEM) algorithm with CT-based attenuation correction; coronal, sagittal, axial, and three-dimensional fusion images were generated using multi-planar reconstruction.

#### Image analysis

Images were reviewed independently by two experienced nuclear medicine physicians. Reviewers evaluated the imaging datasets independently of one another; discrepancies were resolved by consensus and, when necessary, adjudication by a senior nuclear medicine physician. Because this was a retrospective study, formal blinding to all longitudinal clinical information and genotype was not prospectively implemented, and this is acknowledged as a potential source of interpretation bias. Analysis focused on (i) localization of radioactive foci suggestive of residual thyroid tissue in the anterior neck region and (ii) identification of abnormal uptake suggestive of metastasis in cervical or mediastinal lymph nodes, lungs, bones, or other sites. Qualitative interpretation correlated radiotracer uptake with anatomical CT findings (e.g., lesion size, morphology, calcification, and bone destruction); physiological and nonspecific uptake were distinguished from pathological uptake.

### ¹^8^F-FDG PET/CT imaging

#### Instrumentation and imaging protocol

Imaging was performed using a United Imaging uMI 780 digital PET/CT system (United Imaging Healthcare, Shanghai, China), with data processed on a uWI-MI workstation. Patients fasted for at least 6 hours prior to the scan, with blood glucose controlled at ≤ 11.1 mmol/L. ¹^8^F-FDG (radiochemical purity > 95%; Jiangyuan ANTECO Pharmaceuticals, Nanjing, China) was intravenously injected at a dose of 3.70–5.55 MBq/kg. After resting for approximately 60 minutes post-injection and voiding, patients underwent whole-body PET/CT scanning from the skull vertex to the mid-femur in list mode. CT parameters were as follows: tube voltage 120 kV, tube current 100 mA, slice thickness 3 mm, pitch 0.9875. PET acquisition used 3D mode with 4–5 bed positions, 2 minutes per bed position, and a 30% bed overlap.

#### Image analysis

PET images were reconstructed using the OSEM algorithm (2 iterations, 20 subsets) with time-of-flight, point-spread function, and scatter and random corrections. The image matrix was 256 × 256 with 3 mm slice thickness and a 3 mm full-width-at-half-maximum Gaussian filter. CT images were used for attenuation correction and anatomical localization. All fused images were transferred to the uWI-MI workstation for visual and semi-quantitative analysis. Two nuclear medicine physicians with more than 5 years of experience independently reviewed axial, coronal, and sagittal images. A three-dimensional volume of interest was placed at the site of maximum metabolic activity to obtain SUVmax automatically. Background uptake was measured using a 3 cm spherical region of interest in normal right-lobe liver parenchyma. Discrepancies between readers were resolved by a third senior physician. The dual-positive phenotype was assigned at the patient-assessment level as defined above and did not imply that iodine and FDG uptake were co-localized within an identical lesion.

### Molecular testing

#### Nucleic acid extraction

Gene testing was performed on postoperative tumor tissue samples. Total nucleic acid (DNA and RNA) was extracted from formalin-fixed, paraffin-embedded tissue specimens using the QIAamp DNA Mini Kit. DNA and RNA concentrations were quantified using a NanoDrop 1000 spectrophotometer.

### Library preparation and molecular panel

A custom molecular panel was used, targeting single nucleotide variants (SNVs) in 11 genes (*BRAF*, *HRAS*, *KRAS*, *NRAS*, *TP53*, and *TERT* promoter [C228T and C250T]) and fusion variants in 5 genes (RET, *NTRK1*, *NTRK3*, *PAX8*, and *THADA*). Quality control requirements included a total DNA input greater than 10 ng per sample with intact DNA fragment integrity; DNA base Q30 and alignment rates greater than 80% and 90%, respectively; average sequencing depth greater than 500×; RNA target base Q30 rate greater than 80%; and total reads greater than 300,000. Sequencing libraries were prepared using the TruSeq DNA Library Preparation Kit.

### Next-generation sequencing and variant calling

DNA sequencing was performed on an Illumina NextSeq system using paired-end reads (2 × 150 bp). Reads were aligned to the human reference genome GRCh37 using the Burrows-Wheeler Aligner (BWA). MuTect2 and GATK were used to identify SNVs and small insertions/deletions, respectively. All final candidate variants were visually verified using the Integrative Genomics Viewer (IGV).

### Statistical analysis

Continuous data are presented as mean ± standard deviation or median (range), as appropriate; categorical data are presented as count (percentage). Group comparisons were performed using the Fisher exact test, chi-square test, and unpaired t test, as appropriate. The discriminative ability of pre-RAI sTg for RAIR-DTC progression was assessed by receiver operating characteristic (ROC) analysis with calculation of the area under the curve (AUC) and 95% confidence interval. Progression-free survival (PFS) was estimated using the Kaplan-Meier method, with between-group differences evaluated by the log-rank test. Metastatic co-occurrence network analysis was used to describe non-random patterns of metastatic dissemination across anatomical sites in RAIR-DTC. Analyses were performed in SPSS statistical software. A two-sided p value of less than 0.05 was considered statistically significant.

## Results

### RAIR-DTC exhibits a distinct aggressive clinicopathological phenotype

Baseline patient and tumor characteristics are shown in **Table 1** and **Supplementary Table 2**. In the overall DTC cohort, the median age at diagnosis was 44 years, with 72.41% of patients younger than 55 years. Most patients were female (n = 295, 69.58%) and presented with early-stage disease (Stage I–II, n = 349, 82.31%). Tumor multifocality was present in 50.71% of patients, while ETE, vascular invasion, and LNM were observed in 35.38%, 15.80%, and 77.12% of patients, respectively. Nearly all patients received RAI therapy (n = 416, 98.11%), with a median cumulative dose of 100 mCi. A total of 64 patients (15.1%) progressed to RAIR-DTC, including all eight patients who received cumulative doses exceeding 600 mCi.

**Table 1:** Comparison of clinical characteristics between RAIR-DTC and non-RAIR-DTC.

Characteristic	RAIR-DTC (n = 64)	Non-RAIR-DTC (n = 360)	P value
Age at diagnosis			
Median	64	41	<0.001
Range	34-80	16-91	<0.001
<55 years old	13 (20.31)	294 (81.67)	<0.001
≥55 years old	51 (79.69)	66 (18.33)	<0.001
Gender			
Male	22 (34.38)	107 (29.72)	NS
Female	42 (65.63)	253 (70.28)	NS
Gene mutations			
SNV	61 (95.31)	292 (81.11)	<0.01
FA	0 (0.00)	36 (10.00)	<0.01
Negative	3 (4.69)	32 (8.89)	
Tumor size (median, cm)	1.95	1.5	<0.05
Histology			
PTC	54 (84.38)	351 (97.50)	<0.001
cPTC	52 (81.25)	324 (90.00)	<0.001
PTMC	2 (3.13)	0 (0.00)	<0.001
tcvPTC	0 (0.00)	4 (1.11)	<0.001
fvPTC	0 (0.00)	6 (1.67)	<0.001
dsvPTC	0 (0.00)	3 (0.83)	<0.001
ocaPTC	0 (0.00)	1 (0.28)	<0.001
cPTC_fvPTC	0 (0.00)	9 (2.50)	<0.001
cPTC_tcvPTC	0 (0.00)	1 (0.28)	<0.001
cPTC_hvPTC	0 (0.00)	1 (0.28)	<0.001
cPTC_fvPTC_tcvPTC	0 (0.00)	2 (0.56)	<0.001
FTC	9 (14.06)	4 (1.11)	<0.001
ATC	1 (1.56)	0 (0.00)	NS
PTC+FTC	0 (0.00)	1 (0.28)	NS
PTC+PDTC	0 (0.00)	2 (0.56)	NS
Initial sTg (median)	26.6	4.7	NS
Multifocality			
Singleness	41 (64.06)	168 (46.67)	<0.001
Multifocality	23 (35.94)	192 (53.33)	<0.001
ETE	43 (67.19)	107 (29.72)	<0.001
Vascular invasion	36 (56.25)	26 (7.22)	<0.001
LNM	34 (53.13)	293 (81.39)	<0.001
Stage			
I	20 (31.25)	296 (82.22)	<0.001
II	4 (6.25)	29 (8.06)	<0.001
III	4 (6.25)	25 (6.94)	<0.001
IVa	15 (23.44)	0 (0.00)	<0.001
IVb	9 (14.06)	10 (2.78)	<0.001
IVc	12 (18.75)	0 (0.00)	<0.001
AJCC stage			
T stage			
T1	22 (34.38)	206 (57.22)	<0.05
T2	5 (7.81)	40 (11.11)	<0.05
T3	12 (18.75)	73 (20.28)	<0.05
T4	16 (25.00)	41 (11.39)	<0.05
N stage			
N0	22 (34.38)	62 (17.22)	<0.001
N1a	5 (7.81)	117 (32.50)	<0.001
N1b	29 (45.31)	171 (47.50)	<0.001
Nx	8 (12.50)	10 (2.78)	<0.001
M stage			
M0	36 (56.25)	341 (94.72)	<0.001
M1	28 (43.75)	19 (5.28)	<0.001
Metastatic pattern			
SOM	18 (28.13)	16 (4.44)	<0.001
MOM	46 (71.88)	3 (0.83)	<0.001
Cumulative RAI dose (mCi)			
< 600	56 (87.50)	360 (100.00)	<0.001
≥ 600	8 (12.50)	0 (0.00)	<0.001
Recurrence	56 (87.50)	29 (8.06)	<0.001
Number of RAI therapy courses			
<3	52 (81.25)	355 (98.61)	<0.001
≥3	12 (18.75)	5 (1.39)	<0.001

ATC, anaplastic thyroid cancer; cPTC, classical papillary thyroid carcinoma; dsvPTC, diffuse sclerosing variant papillary thyroid carcinoma; ETE, extrathyroidal extension; FA, fusion alteration; FTC, follicular thyroid carcinoma; fvPTC, follicular variant papillary thyroid carcinoma; LNM, lymph node metastasis; mCi, millicurie; MOM, multi-organ metastasis; NS, not significant; non-RAIR-DTC, non-radioiodine-refractory differentiated thyroid cancer; ocaPTC, oncocytic variant papillary thyroid carcinoma; PDTC, poorly differentiated thyroid carcinoma; PTC, papillary thyroid carcinoma; PTMC, papillary thyroid microcarcinoma; RAI, radioactive iodine; RAIR-DTC, radioiodine-refractory differentiated thyroid cancer; SNV, single nucleotide variant; SOM, single-organ metastasis; sTg, serum stimulated thyroglobulin; tcvPTC, tall cell variant papillary thyroid carcinoma.

Continuous variables are presented as median (range) where indicated; categorical variables are presented as n (%). Between-group comparisons used the Fisher exact test, chi-square test, unpaired t test, or Mann-Whitney U test as appropriate. P values are shown as reported in the original analysis; NS, not significant.

Among the 64 RAIR-DTC patients, the most frequent qualifying criterion was Criterion 1 (absence of radioiodine uptake from initial therapy; n = 31, 48.4%), followed by Criterion 4 (disease progression despite iodine uptake; n = 27, 42.2%) and Criterion 3 (spatial heterogeneity of iodine avidity; n = 8, 12.5%). Criterion 2 (prolonged loss of iodine avidity; n = 1) and Criterion 5 (cumulative RAI dose ≥ 600 mCi, frequently co-occurring with other criteria) were less frequent. Based on the correspondence between these qualifying criteria and clinical phenotypes, the RAIR-DTC cases in our cohort grouped into three main phenotypes: a primary non-avid type (predominantly meeting Criterion 1), a heterogeneous or focally avid type (Criterion 3), and a progressing-avid type (Criterion 2 or 4). Detailed case-level distribution is provided in **Supplementary Note 1** and **Supplementary Tables 3** and **4**.

Patients with RAIR-DTC were significantly older at diagnosis compared with the non-RAIR-DTC group (median age 64 vs 41 years; 20.31% vs 81.67% younger than 55 years). Although the primary tumor was larger in the RAIR-DTC group (median 1.95 cm vs 1.5 cm), the LNM rate was significantly lower (53.13% vs 81.39%). Aggressive pathological features were markedly enriched in RAIR-DTC, including significantly higher ETE (67.19% vs 29.72%) and vascular invasion (56.25% vs 7.22%). Histologically, the RAIR-DTC group exhibited a higher prevalence of the more aggressive follicular thyroid carcinoma (14.06%), including one case of anaplastic thyroid carcinoma, whereas these subtypes were rare in non-RAIR-DTC patients (*p* < 0.001). These findings collectively support the highly invasive and dedifferentiated nature of RAIR-DTC tumors.

**Figure 1:**
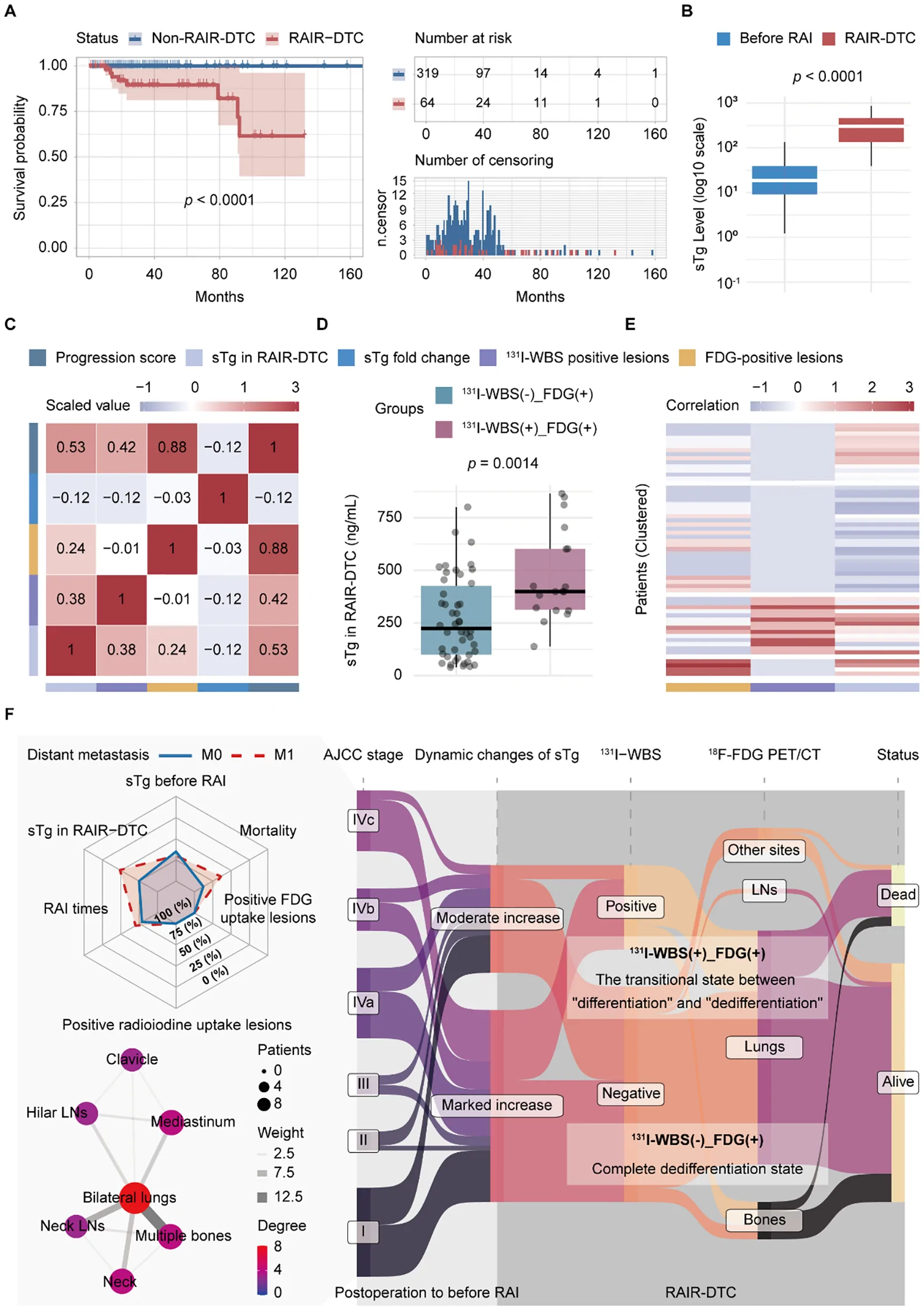
Integrated analysis of clinical, serological, and imaging-correlated features of RAIR-DTC and their associations with patient outcomes. **(A)** Kaplan-Meier survival curves for patients with non-RAIR-DTC versus RAIR-DTC. The number at risk and distribution of censored cases at different time points are shown on the right. **(B)** Comparison of sTg level distributions prior to initial RAI therapy and at the time of RAIR-DTC diagnosis. **(C)** Heatmap depicting correlations among core RAIR-DTC-related indicators. Color intensity corresponds to the strength of the correlation. **(D)** Comparison of log10(sTg) levels in patients with different metastatic phenotypes, including ¹³¹I-WBS(-)/FDG(+) versus ¹³¹I-WBS(+)/FDG(+). **(E)** Clustering heatmap of RAIR-DTC patients based on ¹³¹I-WBS, FDG avidity, and sTg levels at RAIR-DTC diagnosis. **(F)** Sankey diagram illustrating the distribution of sTg dynamics and imaging markers (¹³¹I-WBS, ¹^8^F-FDG PET/CT) across different clinical stages and survival statuses. The radar plot demonstrates the interrelationships among the number of RAI therapy courses, sTg before initial RAI therapy, sTg at RAIR-DTC diagnosis, positive radioiodine uptake lesions, and positive FDG uptake lesions. The metastatic co-occurrence network visualizes non-random metastatic dissemination patterns and locates persistent lesions that retain radioiodine avidity. ¹^8^F-FDG PET/CT, fluorine-18 fluorodeoxyglucose positron emission tomography/computed tomography; ¹³¹I-WBS, iodine-131 whole-body scintigraphy; DTC, differentiated thyroid cancer; FDG, fluorodeoxyglucose; RAI, radioactive iodine; RAIR-DTC, radioiodine-refractory differentiated thyroid cancer; sTg, serum stimulated thyroglobulin.

Disease burden differed substantially between groups. Distant metastasis and multi-organ metastasis (MOM) were significantly more frequent in RAIR-DTC (42.19% vs 5.28%; 71.88% vs 0.83%; both *p* < 0.001). Consistently, Stage IV disease was significantly more common in RAIR-DTC, with Stage IVa (locally advanced) and IVc (distant metastatic) observed exclusively in this cohort. Despite cumulative RAI activities remaining below 600 mCi in most RAIR-DTC cases (87.50%), recurrence was markedly more common (87.50% vs 8.06%). The proportion of patients receiving ≥3 treatment courses was also higher (18.75% vs 1.39%), reflecting the refractory nature of RAIR-DTC. Notably, survival was significantly reduced in RAIR-DTC (*p* < 0.0001; **Figure 1A**).

### Dynamic sTg elevation reveals non-linear evolution of dual-modal imaging phenotypes

sTg is an established marker for postoperative monitoring in DTC, and its level at the time of initial RAI therapy can indicate the presence of metastases prior to RAI administration. To evaluate sTg as a quantitative indicator of disease evolution, we analyzed its longitudinal trajectory from initial RAI treatment to RAIR-DTC diagnosis. Median sTg levels increased significantly from 18.7 ng/mL to 302.7 ng/mL during this progression (*p* < 0.001; **Figure 1B**). At the time of RAIR-DTC diagnosis, sTg levels demonstrated consistent positive associations with the number of both radioiodine-avid and FDG-avid lesions; disease burden scores also correlated positively with FDG-avid lesion counts (**Figure 1C**).

Assessment of the predictive value of sTg demonstrated excellent discrimination for RAIR-DTC progression. Pre-RAI sTg achieved an AUC of 0.911 (95% CI 0.856–0.967; **Supplementary Figure 2A**), with an optimal cutoff value of 10.77 ng/mL. Comparison with previously reported thresholds revealed that this cutoff lies above values typically reported in low- or intermediate-risk DTC cohorts, yet remains within the range described in high-risk or metastatic populations (**Supplementary Figure 3**; **Supplementary Table 5**), supporting its relevance for RAIR-DTC risk stratification.

Next, we analyzed the association between the average number of iodine-avid and FDG-avid lesions across different imaging subgroups (**Supplementary Figure 2B**). In the ¹³¹I-WBS(-)/FDG(+) phenotype, higher sTg levels were associated with a greater FDG-avid lesion burden (patients with sTg > 500 ng/mL had approximately 4.5 FDG-avid lesions). In contrast, in the ¹³¹I-WBS(+)/FDG(+) phenotype, iodine-avid and FDG-avid lesion counts increased concurrently (patients with sTg > 500 ng/mL had approximately 1.5 iodine-avid lesions and 3 FDG-avid lesions). RAIR-DTC has traditionally been viewed as a complete switch from “radioiodine affinity” to “FDG affinity” patterns. However, our findings indicate that this process is heterogeneous and progressive rather than binary. The dual-positive group appeared predominantly in the intermediate (101–500 ng/mL) and high (> 500 ng/mL) sTg strata. In contrast, the ¹³¹I-WBS(-)/FDG(+) group appeared across all sTg strata, particularly in the extremely high stratum (> 500 ng/mL) and the low stratum (≤ 100 ng/mL), implying extensive and early dedifferentiation (**Supplementary Figure 2C**). Notably, density contour plots revealed that iodine-avid lesion counts increased proportionally with sTg levels and were primarily concentrated within the intermediate and high sTg range (**Supplementary Figure 2D**).

sTg levels varied substantially with metastatic progression patterns. Dual-positive metastatic lesions exhibited higher sTg levels compared with FDG-only lesions (*p* = 0.0014; **Figure 1D**). Clustering of RAIR-DTC cases based on standardized ¹³¹I-WBS, sTg, and FDG metrics identified distinct subgroups with coordinated imaging-biomarker profiles (**Figure 1E**). Longitudinal analyses further confirmed dynamic genotype-phenotype relationships. Patients with postoperative Stage IVc disease exhibited the highest FDG positivity and elevated sTg levels (**Figure 1F**, Sankey diagram and radar plot). Non-survivors had greater sTg fold-change and more FDG(+) lesions compared with survivors. Across metastatic sites, bilateral lung or multiple bone metastases were associated with significantly higher sTg levels and FDG positivity. Furthermore, patients receiving ≥3 RAI therapies exhibited declining ¹³¹I-WBS positivity alongside increasing FDG positivity, reflecting progressive tumor dedifferentiation with repeated RAI treatments.

Metastatic co-occurrence network analysis further revealed non-random dissemination patterns in RAIR-DTC (**Figure 1F**, metastatic co-occurrence network). Cervical lymph nodes and bilateral lungs acted as central hubs, with the mediastinum frequently involved, and disease spread in an orderly fashion from these hubs to other sites via lymphatic and hematogenous routes. This pattern provides a potential explanation for the distribution of metastatic sites and highlights persistent lesions that may progress more slowly and retain iodine avidity.

Collectively, these findings support the notion that dedifferentiation in RAIR-DTC is a progressive, regional process and establish sTg as a quantitative indicator of disease evolution and metastatic burden.

### *TERT* promoter mutation is associated with aggressive molecular and clinical features in RAIR-DTC

**Figure 2:**
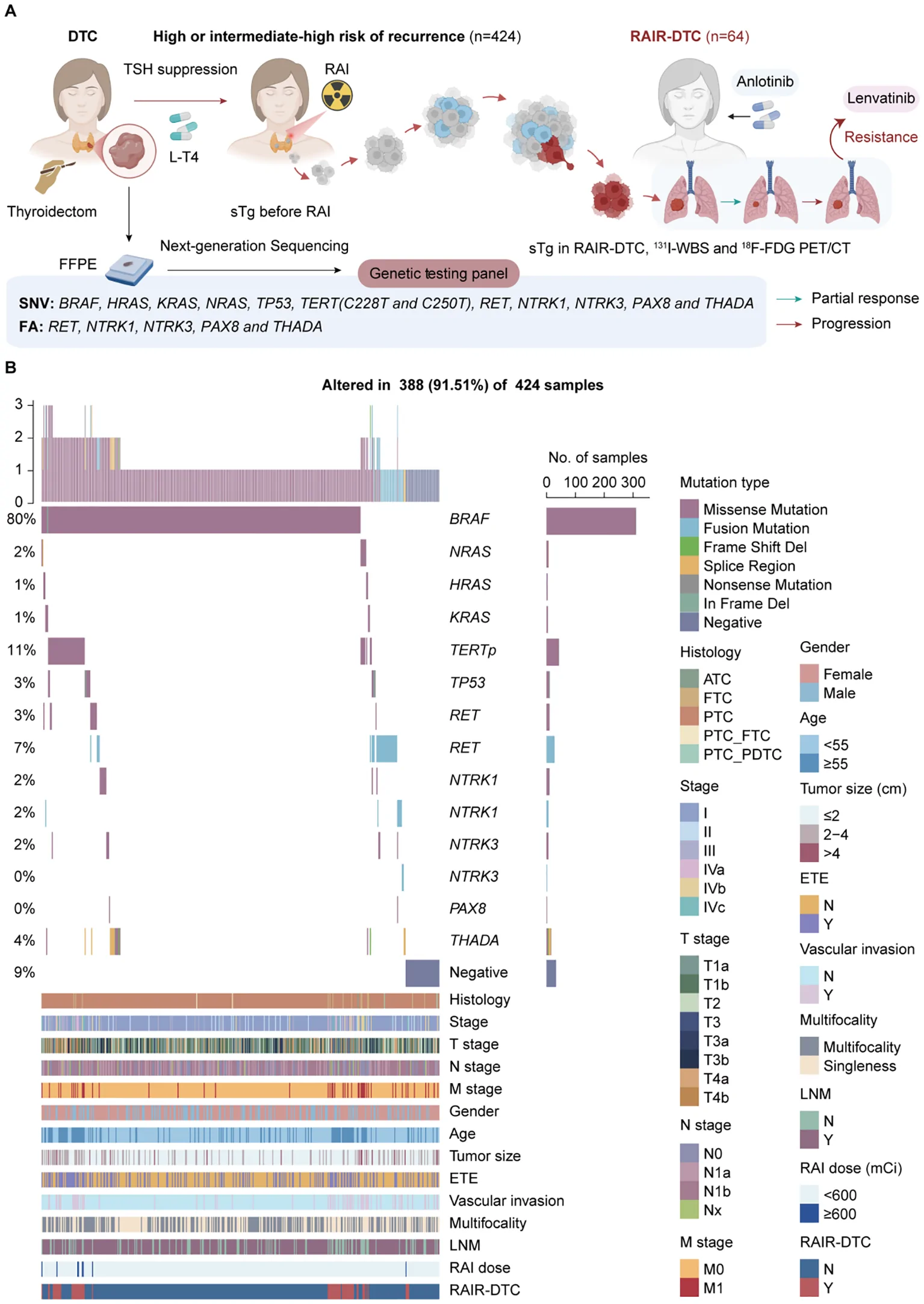
Clinical trajectories and genomic landscape of the DTC cohort. **(A)** Trajectories of clinical management and disease progression from DTC to RAIR-DTC in our cohort. **(B)** Alteration frequencies of key DTC driver genes, including *BRAF*, the RAS family, *TERT*, *TP53*, RET, *NTRK1*/3, *PAX8*, *THADA*, and negative cases. Alterations are annotated by mutation type and correlated with histological subtypes. The heatmap shows associations between genetic alterations and clinicopathological variables. DTC, differentiated thyroid cancer; RAIR-DTC, radioiodine-refractory differentiated thyroid cancer.

To characterize the molecular features associated with progression to RAIR-DTC, we first examined the genomic landscape of the overall DTC cohort (**Figure 2**; **Supplementary Table 2**). Among 424 tumor samples from patients with DTC, genetic alterations were detected in 388 cases (91.51%). The most frequently mutated genes were *BRAF* (80%) and *TERTp* (11%), while mutations in *TP53*, RET, *NRAS*, *HRAS*, *KRAS*, and members of the NTRK family were detected at lower frequencies (1–3%). *BRAFV600E* mutations were the most prevalent across all histological subtypes, particularly in papillary thyroid carcinoma (95.52%). Most tumors harbored a single detectable variant, with missense mutations constituting the predominant alteration type (> 80%), followed by frameshift deletions and nonsense mutations. Among single nucleotide variants (SNVs), T>A transversions were the most common (n = 349), followed by C>T (n = 86), T>C (n = 16), C>G (n = 7), and C>A (n = 6) (**Supplementary Figure 1A**).

We further investigated the association between mutations and disease recurrence (**Supplementary Figure 1B**). Mutation frequencies were largely comparable between the recurrence group (n = 80) and the non-recurrence group (n = 308) across most genes, including *BRAF* (92% vs 95%), *TP53* (1% vs 4%), RET (3% vs 3%), and *NRAS* (4% vs 1%). In contrast, *TERTp* mutation rate was significantly higher in the recurrence group (40% vs 6%, *p* < 0.0001). Concordantly, patients harboring *TERTp* mutation exhibited significantly reduced PFS (*p* < 0.0001), supporting *TERTp* mutation as a key molecular driver of poor prognosis in DTC (**Supplementary Figure 1C**).

**Figure 3:**
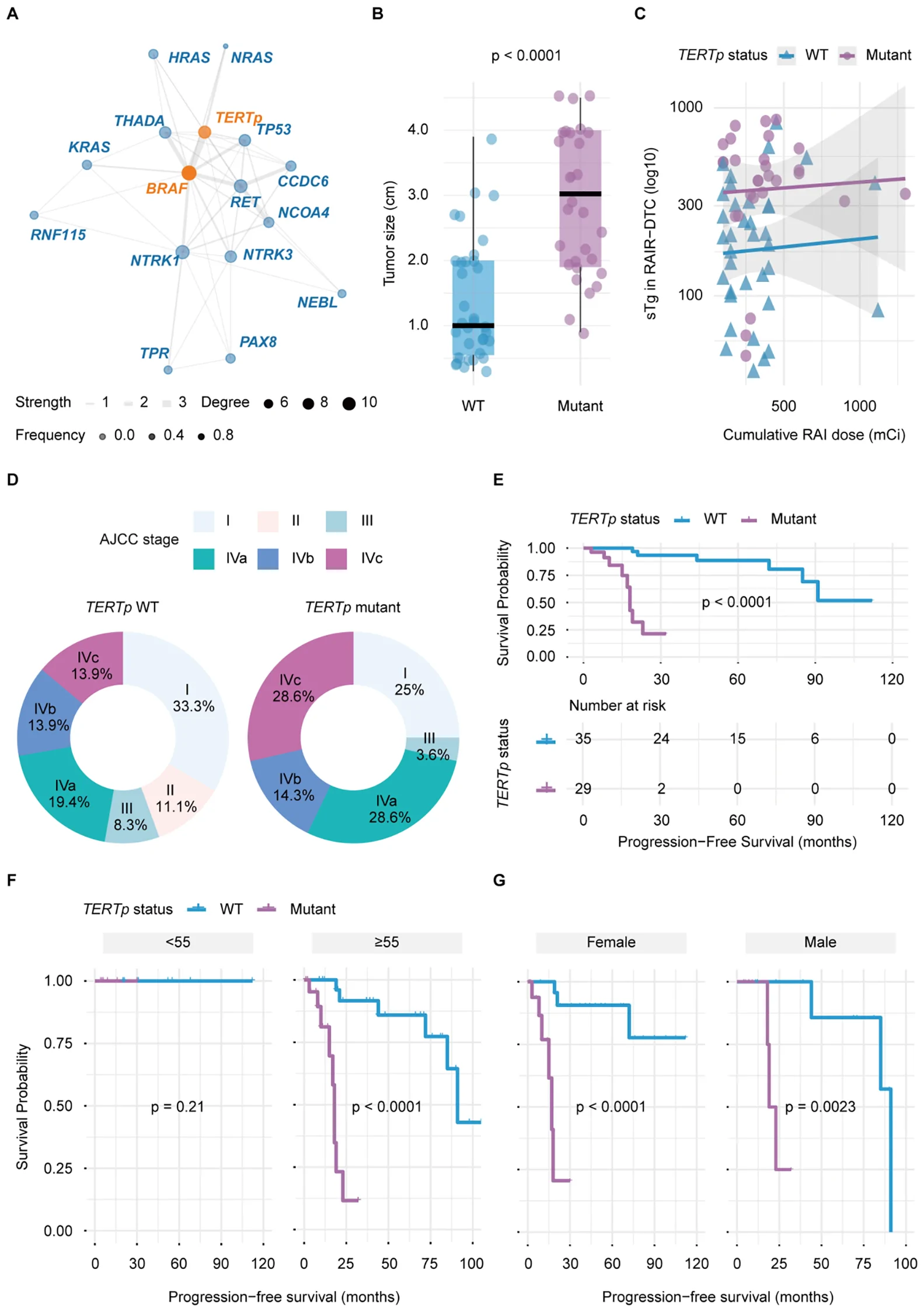
*TERTp* mutation correlates with adverse clinical outcomes and distinct molecular characteristics in RAIR-DTC. **(A)** Gene co-occurrence network analysis showing interactions between *TERTp* and key oncogenes (e.g., *BRAF*, *HRAS*, *NRAS*, *KRAS*) and tumor suppressors (e.g., *TP53*) in DTC. Node size represents connectivity degree, node color indicates mutation frequency (orange = high, blue = low), and edge thickness reflects interaction strength. **(B)** Box plot comparing tumor size between *TERTp* WT and mutant groups (*p* < 0.0001). **(C)** Scatter plot showing the relationship between cumulative RAI dose and sTg levels in *TERTp* WT and mutant groups. **(D)** Donut charts illustrating AJCC stage distribution in *TERTp* WT and mutant groups. *TERTp*-mutant group had a higher proportion of advanced-stage disease (e.g., IVc 28.6% vs 13.9% in WT). **(E)** Kaplan-Meier analysis of PFS comparing *TERTp* wild-type and mutant RAIR-DTC (*p* < 0.0001). The number of patients at risk is shown below the curve. **(F)** Age-stratified Kaplan-Meier PFS analysis (< 55 vs ≥ 55 years). *TERTp* mutation was associated with significantly worse PFS in the ≥ 55 years subgroup (*p* < 0.0001). **(G)** Sex-stratified Kaplan-Meier PFS analysis. *TERTp* mutation correlated with poorer PFS in both sexes. AJCC, American Joint Committee on Cancer; DTC, differentiated thyroid cancer; PFS, progression-free survival; RAI, radioactive iodine; RAIR-DTC, radioiodine-refractory differentiated thyroid cancer; sTg, serum stimulated thyroglobulin; *TERTp*, *TERT* promoter; WT, wild-type.

We next compared the genomic alteration landscapes between RAIR-DTC and non-RAIR-DTC (**Table 1**). The proportion of mutation-negative cases (4.69%) was lower in the RAIR-DTC group than in the non-RAIR-DTC group (8.89%). SNVs were detected more frequently in patients with RAIR-DTC (95.31% vs 81.11%), yet fusion alterations were detected exclusively in non-RAIR-DTC cases (RET fusions 7.78%, NTRK fusions 2.22%). Core driver mutations differed substantially between groups, including a higher prevalence of *BRAFV600E* mutations (87.50% vs 78.89%) and *TERTp* mutations (43.75% vs 5.28%, *p* < 0.001). Mutation co-occurrence network analysis further positioned *TERTp* as a central molecular hub (**Figure 3A**), which was closely connected with key oncogenic drivers (e.g., *BRAFV600E*, *HRAS*, *NRAS*, *KRAS*) and tumor suppressor genes (e.g., *TP53*). Notably, *BRAFV600E* exhibited the highest connectivity with *TERTp*, highlighting a synergistic oncogenic network centered on *TERTp* activation and MAPK pathway dysregulation.

Clinically, *TERTp* mutations were consistently associated with aggressive clinicopathological characteristics. Age at diagnosis and sex distribution were comparable between groups; however, tumor size was significantly larger in the *TERTp*-mutant group (median 3.1 cm vs 1.0 cm, *p* < 0.0001) (**Figure 3B**), accompanied by a higher rate of LNM (67.86% vs 41.67%, *p* < 0.05). Patients with *TERTp*-mutant RAIR-DTC had persistently higher sTg levels across increasing cumulative RAI doses (**Figure 3C**), indicating refractory disease despite intensive RAI therapy, consistent with the higher proportion of *TERTp*-mutant patients requiring ≥3 RAI courses (26.7% vs 9.1% in wild-type, *p* = 0.03). Patients with *TERTp*-mutant RAIR-DTC exhibited more advanced disease, characterized by higher prevalence of Stage IVc disease (28.6% vs 13.9% in wild-type) (**Figure 3D**), T4 stage (60.71% vs 16.67%, *p* < 0.001), and N1b stage (57.14% vs 36.11%, *p* < 0.05). MOM at RAIR-DTC diagnosis was more frequent among patients harboring *TERTp* mutations (82.14% vs 33.33%, *p* < 0.001). Recurrence was observed in all *TERTp*-mutant cases (100% vs 77.78%, *p* < 0.001), reinforcing the association between *TERTp* mutation and aggressive, treatment-resistant disease (**Supplementary Table 6**). Concordantly, *TERTp* mutation was associated with significantly shorter PFS (median PFS 18.2 vs 47.6 months; log-rank *p* < 0.0001) (**Figure 3E**). This association was particularly pronounced in patients aged ≥55 years (*p* < 0.0001) (**Figure 3F**) and was evident in both sexes, with a greater effect in females (*p* < 0.0001 for females vs *p* = 0.0023 for males) (**Figure 3G**).

A high co-mutation rate of *BRAFV600E*/*TERTp* was observed in our RAIR-DTC cohort, with 38.1% of patients with *TERTp* mutation also harboring *BRAFV600E* (**Supplementary Table 7**). Patients harboring *BRAFV600E*/*TERTp* co-mutation were significantly older and had larger tumors than those with *BRAFV600E* mutation alone (both *p* < 0.0001) (**Supplementary Figures 4A, C**). Moreover, the proportion of patients receiving cumulative RAI dose ≥600 mCi was higher in the co-mutation group (*p* < 0.01), consistent with more aggressive disease and poorer treatment response. Patients with *BRAFV600E*/*TERTp* co-mutation showed higher rates of N1b-stage LNM (66.67% vs 34.48%, *p* = 0.011), MOM (100% vs 58.62%, *p* < 0.001), and recurrence (100% vs 75.9%, *p* = 0.012). Among patients with tumor stages III–IV, both the proportion who received a cumulative RAI dose ≥600 mCi and the sTg level at the time of RAIR-DTC diagnosis were significantly higher among those with *BRAFV600E*/*TERTp* co-mutation than among those with *BRAFV600E* mutation alone (*p* < 0.01) (**Supplementary Figures 4B, D, E**), indicating that despite receiving more intensive RAI therapy due to greater disease aggressiveness, patients with co-mutation had worse treatment outcomes. Survival analysis further demonstrated significantly worse PFS in patients with *BRAFV600E*/*TERTp* co-mutation (*p* < 0.01) (**Supplementary Figure 4F**).

### *BRAFV600E*/*TERTp* co-mutation is associated with metabolic hyperactivity and targeted therapy resistance in RAIR-DTC

**Figure 4:**
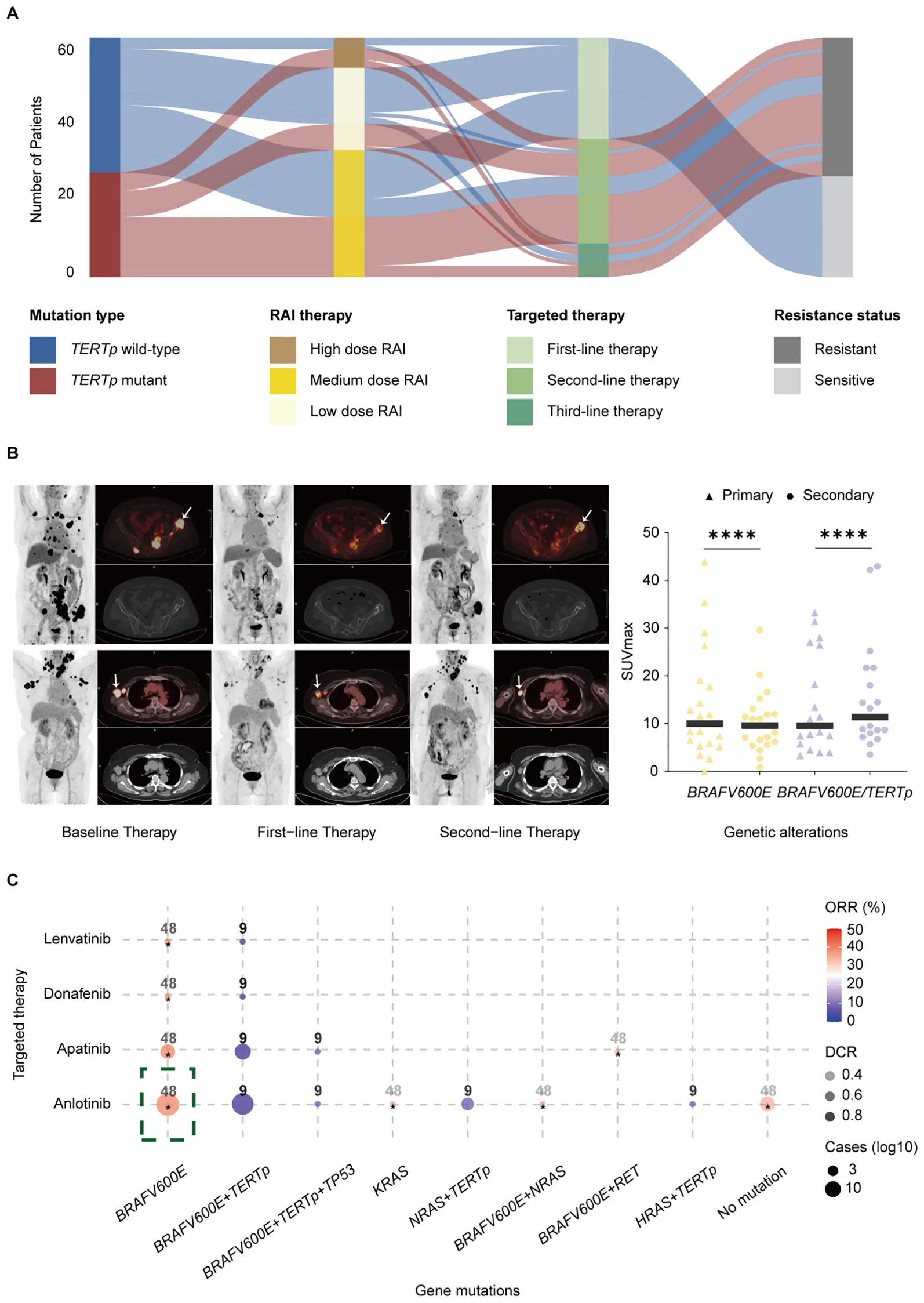
*TERTp* mutation impacts treatment sequencing, ¹^8^F-FDG metabolic phenotype, and targeted therapy responses in RAIR-DTC. **(A)** Treatment trajectory plot tracking patient cohorts across four stages: mutation type (*TERTp* wild-type/mutant), RAI therapy dose (high/medium/low dose), targeted therapy line (first/second/third line), and resistance status (resistant/sensitive); band width corresponds to the number of patients at each stage. **(B)** Representative ¹^8^F-FDG PET/CT images of metastatic lesions at baseline, first-line targeted therapy, and second-line targeted therapy (arrows denote metastatic lesions). Box-and-whisker plot of maximum standardized uptake value (SUVmax) for primary (▲) and secondary (•) lesions, stratified by genetic alterations (*BRAFV600E* vs *BRAFV600E*/*TERTp*). **(C)** Bubble plot of targeted therapy efficacy by gene mutation subtype. ¹^8^F-FDG PET/CT, fluorine-18 fluorodeoxyglucose positron emission tomography/computed tomography; PET, positron emission tomography; RAI, radioactive iodine; RAIR-DTC, radioiodine-refractory differentiated thyroid cancer; SUVmax, maximum standardized uptake value; *TERTp*, *TERT* promoter; WT, wild-type.

We next dissected the relationships among genotype, metabolic imaging phenotype, and targeted therapy response in RAIR-DTC (**Figure 4A**). Among patients receiving first-line tyrosine kinase inhibitors (TKIs), radiographic progression within 6 months occurred more frequently in *TERTp*-mutant cases compared with wild-type counterparts (68.4% vs 23.5%, *p* = 0.001). Patients harboring *TERTp* mutations were more likely to receive higher cumulative RAI doses and multi-line targeted therapies, whereas wild-type cases more frequently remained responsive to earlier-line treatments.

We further assessed the relationship between genotype and metabolic imaging phenotype (**Figure 4B**). Representative ¹^8^F-FDG PET/CT images of RAIR-DTC cases demonstrated persistent metabolically active lesions. Lesions from patients harboring BRAFV600E/TERTp co-mutations showed higher observed SUVmax than those from patients with BRAFV600E alone (p < 0.0001), irrespective of lesion origin. We interpret this finding as an association with a hypermetabolic phenotype rather than as proof of genotype-driven metabolic aggressiveness. SUVmax is influenced by lesion size and partial-volume effects, scanner/reconstruction characteristics, uptake conditions, blood glucose, and the endocrine/TSH context at imaging. In particular, SUVmax may be underestimated in smaller lesions because of limited spatial resolution. Lesion-level multivariable adjustment for these factors was not available in the present retrospective dataset; therefore, residual confounding remains possible. Targeted therapy efficacy also varied across genetic subgroups (**Figure 4C**). For instance, anlotinib achieved a higher objective response rate in the BRAFV600E subgroup, whereas reduced efficacy was observed in the co-mutation or RAS-mutated cohorts.

These observations support integrating *TERTp* mutation status with dynamic sTg-imaging phenotyping to guide personalized therapeutic decisions in RAIR-DTC.

## Discussion

This study integrated longitudinal sTg kinetics, dual-modal imaging phenotypes, and genomic profiling to characterize the dynamic progression of RAIR-DTC and the molecular determinants of targeted therapy resistance. Moving beyond traditional static or single-gene analyses, our work delineates an evolving sTg-imaging phenotype framework, demonstrates the central role of *TERTp* mutation, particularly in co-occurrence with *BRAFV600E*, in promoting metabolic aggressiveness and TKI resistance, and clarifies metastatic dissemination patterns relevant to surveillance strategy.

### sTg kinetics as a dynamic biomarker coupled to evolving dual-modal imaging phenotypes

Dynamic changes in sTg have been implicated in metastatic progression and survival in DTC. In our cohort, greater sTg fold-changes were observed in patients with adverse outcomes, consistent with a link between sTg kinetics and disease aggressiveness rather than tumor burden alone. In addition, patients with bilateral lung or multiple bone metastases had significantly higher sTg levels and FDG positivity. These sites correspond to dominant dissemination patterns in RAIR-DTC and align with the metastatic co-occurrence network, suggesting that rising sTg levels may capture coordinated processes of metabolic activation and organ-specific metastatic progression.

A key contribution of this study is the establishment of a dynamic sTg threshold along the RAIR-DTC progression trajectory. sTg levels increased nonlinearly from initial RAI therapy to RAIR-DTC diagnosis, and an sTg level greater than 10.77 ng/mL demonstrated strong discriminatory performance for predicting RAIR transformation. Previous studies primarily focused on predicting recurrence (with cutoffs typically ranging from 1.0–17.8 ng/mL), while thresholds reported specifically for RAIR-DTC (e.g., 49.2 ng/mL) are often used to confirm late-stage refractory disease (9–16). Our threshold lies within the transitional range, potentially identifying the critical window between preserved RAI responsiveness and progressive dedifferentiation. We also systematically revealed a dynamic stratification between sTg levels and dual-modal imaging phenotypes, with distinct phenotypic correlations across sTg strata. This nonlinear pattern challenges the simple linear dedifferentiation model, indicating that RAIR-DTC evolution is heterogeneous and dynamic, likely involving the selection and evolution of different clonal subpopulations.

Mechanistically, the differences in imaging phenotypes across sTg strata may reflect underlying differences in differentiation status, metabolic reprogramming, and pathway activation patterns (9, 15, 17). In the early stages of dedifferentiation (sTg ≤ 100 ng/mL), MAPK pathway activation likely drives the loss of NIS function while partially preserving Tg synthesis, resulting in the ¹³¹I-WBS(-)/FDG(+) phenotype. At the intermediate stage (sTg 101–500 ng/mL), co-activation of PI3K/Akt/mTOR and MAPK pathways may maintain some iodine uptake and stronger Tg secretion (4), as reflected in the dual-positive phenotype and high intratumoral heterogeneity. The high sTg stage (> 500 ng/mL) is likely driven by *TERTp* mutations, which suppress differentiation programs through epigenetic reprogramming (e.g., inhibiting differentiation regulators such as TTF-1) (18). In this stage of deep dedifferentiation, FDG positivity peaks and glycolytic metabolism becomes dominant, corresponding to more metastatic lesions in the ¹³¹I-WBS(-)/FDG(+) phenotype than in the ¹³¹I-WBS(+)/FDG(+) phenotype. Despite increased tumor burden, sTg elevation is limited, likely due to impaired thyroglobulin synthesis capacity.

The metastatic co-occurrence network provides practical guidance for clinical metastatic screening. Patients with low sTg levels should prioritize cervical and pulmonary micrometastasis screening via ¹^8^F-FDG PET/CT. Patients with intermediate sTg levels require combined dual-modal imaging to evaluate mixed lesions in cervical lymph nodes, hilar lymph nodes, and mediastinum. Patients with high sTg levels need comprehensive evaluation of distant metastases related to hematogenous spread. Moreover, patients with disease progression after ≥3 RAI courses should switch to ¹^8^F-FDG PET/CT monitoring promptly and consider alternative therapies such as targeted therapy.

Notably, the positive correlation between sTg fold-change and the number of FDG-positive lesions provides an indicator for early identification of dedifferentiation. Even with low absolute sTg, a significant short-term doubling should prompt suspicion of dedifferentiation and timely ¹^8^F-FDG PET/CT evaluation. This addresses the limitation of traditional static indicators and supports dynamic sTg monitoring as a key strategy for RAIR-DTC risk stratification.

In summary, integrating dynamic sTg changes with dual-modal imaging data enabled the development of an sTg-imaging phenotype framework for RAIR-DTC. This framework refines disease monitoring strategies across progression stages and provides a biologically informed basis for risk-adapted clinical management.

### *TERTp* mutation is associated with aggressiveness and targeted therapy resistance in RAIR-DTC

In our RAIR-DTC cohort, patients exhibited a distinct genomic profile dominated by high-frequency *BRAF* and *TERTp* mutations, consistent with previous findings (19). Beyond confirming the prognostic role of *TERTp* described in earlier reports, our cohort uniquely links genotype to ¹^8^F-FDG metabolic phenotype and real-world TKI response trajectories. Notably, these genetic alterations consisted exclusively of SNVs, particularly *TERTp* mutations, with no fusion alterations detected. This observation contrasts with a previous report on advanced thyroid cancer, which reported NTRK fusion frequencies of approximately 4% (20). Our findings instead suggest that a point mutation-driven pattern, represented by *TERTp*, is the primary molecular driver in RAIR-DTC. This aligns with previous work in which *TERTp* mutations were associated with more aggressive clinical phenotypes and therapy resistance (21). These observations support the view that *TERTp* mutations and concurrent genetic alterations (e.g., TP53 mutations) may collectively drive disease progression and refractoriness.

More importantly, our analysis revealed the synergistic effect of *TERTp* co-mutation with *BRAFV600E*. Patients carrying the co-mutation exhibited more extensive LNM, near-universal MOM and recurrence, higher cumulative RAI doses, and poorer prognosis. The co-occurrence of these drivers acts as a molecular “second hit” that exacerbates disease aggressiveness and treatment resistance. Mechanistically, while *BRAFV600E* mutation downregulates NIS expression via the MAPK pathway, thereby reducing iodine uptake capacity, *TERTp* mutation may further inhibit NIS membrane localization through epigenetic reprogramming and enhance tumor cell DNA damage repair capacity, reducing the cytotoxicity of ¹³¹I. These complementary mechanisms provide a biologically plausible explanation for the pattern of increased treatment intensity yet limited therapeutic benefit observed in our cohort. Furthermore, the persistently elevated sTg levels observed in patients with co-mutation across disease stages and cumulative RAI doses support the association between *TERTp* mutation and a high burden of metabolically active, poorly differentiated tumor clones. This is consistent with our finding that co-mutated lesions exhibit higher SUVmax on ¹^8^F-FDG PET/CT (**Figure 4B**), linking genotype to a hypermetabolic phenotype.

Stratified analysis by tumor stage showed that the adverse prognostic effect of *TERTp* co-mutation was more pronounced in advanced RAIR-DTC, consistent with the higher tumor heterogeneity and more pronounced dedifferentiation at this stage. *TERTp* mutation-associated telomerase activation may sustain proliferative capacity in dedifferentiated tumor cells, thereby facilitating disease progression, angiogenesis, and metastatic dissemination (22). In contrast, earlier-stage tumors typically retain a higher degree of differentiation, and the phenotypic consequences of *TERTp* mutation may be less pronounced, potentially attenuating detectable survival differences. These findings have direct implications for clinical risk stratification: in patients with stage III–IV *BRAFV600E*-mutant DTC, evaluation of *TERTp* mutation status may help identify individuals at elevated risk for developing aggressive disease.

From the perspective of nuclear medicine clinical practice, these findings emphasize the importance of integrating molecular testing with imaging evaluation. Patients harboring *BRAFV600E*/*TERTp* co-mutations exhibited a more aggressive clinical course, characterized by a higher propensity for MOM, particularly involving the lungs and bones. ¹^8^F-FDG PET/CT has significantly higher sensitivity than ¹³¹I-WBS for detecting such hypermetabolic metastatic foci, and should therefore be prioritized for metastatic screening and treatment response evaluation in this subgroup.

From a clinical standpoint, assessment of *TERTp* mutation status may further refine risk stratification among patients with *BRAFV600E*-mutant DTC at intermediate- to high-risk. Early consideration of targeted therapies (e.g., MAPK pathway inhibitors) is warranted in this population, rather than repeat empiric RAI treatments. Furthermore, patients harboring *BRAFV600E*/*TERTp* co-mutations exhibited less favorable responses to certain targeted therapies such as anlotinib and lenvatinib. Future research could explore specific therapeutic strategies targeting *TERTp* mutations, including combinations targeting telomerase activity, MAPK pathway inhibition, or immune modulation, to improve outcomes in this high-risk population.

Importantly, when interpreting the association between BRAFV600E/TERTp co-mutation and higher SUVmax, several technical and biological confounders inherent to retrospective PET/CT analysis warrant consideration. SUVmax is a semi-quantitative parameter influenced by multiple factors beyond true metabolic activity. Partial volume effects can lead to substantial underestimation of SUVmax in small lesions (<2–3 times the spatial resolution of the PET system), potentially attenuating or confounding genotype-metabolism correlations if lesion sizes differ systematically between genetic subgroups. Although we observed larger primary tumors in the co-mutation group, the distribution of metastatic lesion sizes was not uniformly available for lesion-level adjustment, and we cannot exclude the possibility that the higher SUVmax partly reflects a larger lesion burden rather than intrinsically higher glucose metabolism per unit of viable tumor tissue. Furthermore, the timing of PET/CT relative to the last RAI therapy, TSH levels at the time of scanning (which can stimulate residual thyroid tissue and potentially alter FDG avidity in differentiated components), and serum glucose levels are well-recognized modulators of FDG uptake. While our clinical protocol encouraged consistent pre-scan preparation (≥6-hour fast, blood glucose ≤11.1 mmol/L), TSH was not uniformly suppressed to the same target before all scans due to the real-world retrospective design, and TSH variability may have introduced noise into SUVmax comparisons. These factors, together with known scanner/reconstruction-related variability, underscore that the observed SUVmax difference between genotypes should be interpreted as a robust association with a hypermetabolic imaging phenotype, rather than a direct quantitative measure of genotype-driven metabolic aggressiveness. Prospective studies with standardized imaging protocols, lesion-size matching, and multivariable adjustment for TSH and other metabolic confounders are needed to confirm the independent contribution of TERTp status to tumor glycolytic phenotype.

Our results demonstrate that *BRAFV600E*/*TERTp* co-mutation is consistently associated with treatment resistance and poor prognosis in RAIR-DTC, with a more pronounced effect in advanced disease. These observations provide a biologically grounded rationale for incorporating *TERTp* status into risk-assessment frameworks and may inform imaging surveillance strategies and therapeutic decision-making in nuclear medicine practice.

### Limitations

This study has several limitations. First, its retrospective design introduces the possibility of selection and referral bias. Inclusion required sufficiently complete RAI, serologic, imaging, molecular, and follow-up data; therefore, patients with incomplete records were excluded and the analyzed cohort may overrepresent patients undergoing more intensive surveillance. Missing data were handled through complete-case analysis for the corresponding analyses rather than by prospective standardized imputation, and this may introduce additional bias. Second, although post-therapeutic ¹³¹I-WBS was standardized at 4 days after RAI, ¹^8^F-FDG PET/CT and other cross-sectional examinations were obtained according to clinical indications rather than at fixed longitudinal intervals. This heterogeneity may affect the apparent timing of phenotype transitions and lesion detection. Imaging was independently interpreted by experienced nuclear medicine physicians, but formal prospective blinding to all clinical information and genotype was not implemented. Third, sTg is assay dependent. Two immunoassay platforms were used during the study period; despite a reported analytical sensitivity of 0.04 ng/mL and exclusion of six patients with elevated TgAb from sTg-based analyses, no formal inter-platform calibration was performed and functional sensitivity was not independently determined in this cohort. Consequently, the 10.77 ng/mL cutoff should be regarded as cohort-specific and requires external assay-specific validation. Fourth, SUVmax comparisons may be influenced by partial-volume effects, lesion size, acquisition/reconstruction factors, blood glucose, and TSH/endocrine conditions. Because lesion-level multivariable adjustment for these potential confounders was not available, the higher SUVmax observed in BRAFV600E/TERTp co-mutated tumors should be interpreted as an association with a hypermetabolic phenotype rather than a causal measure of genotype-driven metabolic aggressiveness. Fifth, the study is primarily based on a Chinese population, and the generalizability of the sTg-imaging phenotype framework across different ethnicities and healthcare settings requires further evaluation. Finally, while the targeted panel covers major thyroid-cancer drivers, broader whole-exome or transcriptome analyses may reveal additional molecular determinants of dedifferentiation and TKI resistance.

## Conclusion

Coupling longitudinal sTg kinetics with evolving dual-modal imaging phenotypes delineates genotype-associated disease courses in RAIR-DTC. *TERT* promoter mutation, particularly when co-occurring with *BRAFV600E*, characterizes a metabolically aggressive, treatment-resistant subset marked by elevated sTg dynamics, predominantly FDG-avid metastatic phenotypes, higher SUVmax, and early progression on first-line TKIs. These findings support a dynamic phenotyping approach for refining surveillance strategies and informing genotype-guided treatment decisions in RAIR-DTC, and provide a rationale for prospective studies to evaluate phenotype-genotype-stratified management.

## Data Availability

The data generated in this study are publicly available in the NCBI BioProject database, accession number PRJNA1427906.
