# Supplementary material for: Dynamic phenotyping of radioiodine-refractory differentiated thyroid cancer: thyroglobulin trajectories, dual-modal imaging, and genotype-defined treatment resistance
Source: Front Endocrinol (Lausanne). 2026 Aug 10;17:1891291. doi: 10.3389/fendo.2026.1891291 (PMC13520594; doi:10.3389/fendo.2026.1891291)
Supplement: Supplementary file 1 [file SupplementaryFile1.docx]

**Supplementary Note 1. Distribution of RAIR-DTC diagnostic criteria in the cohort.**

The diagnosis of RAIR-DTC was established based on five non-mutually exclusive criteria. Thirty-one patients (48.4%) met Criterion 1 (absence of radioiodine uptake from initial therapy), representing the largest subgroup. These patients demonstrated no iodine uptake in metastatic lesions on ¹³¹I-WBS following initial treatment, while disease presence was confirmed by ¹⁸F-FDG PET/CT. This pattern suggests that metastatic lesions in these patients may have been dedifferentiated or non-avid from the outset. Representative cases include P1, P2, and P6. The aggressive clinical course of this subgroup was further evidenced by marked thyroglobulin elevation or death in cases such as P12 and P27.

Criterion 4 (disease progression despite iodine uptake) was met by 27 patients (42.2%). Key characteristics included clear radioiodine uptake on ¹³¹I-WBS accompanied by a sharp rise in serum thyroglobulin levels within one year or the appearance of new lesions. This subgroup (e.g., P4, P9, P34) represents tumors that retain iodine avidity but respond poorly to RAI therapy.

Eight patients met Criterion 3, characterized by spatial heterogeneity where some lesions remained iodine-avid while others were non-avid, with ¹⁸F-FDG PET/CT showing high metabolic activity (e.g., P3, P15, and P47). This subgroup underscores the critical role of ¹⁸F-FDG PET/CT in identifying such heterogeneity.

Criterion 2 and 5 were less frequent in this cohort. Only one patient (P20) met Criterion 2, with a prolonged disease course spanning decades, illustrating the slow evolution of RAIR and the gradual loss of iodine avidity. Criterion 5 (cumulative dose ≥ 600 mCi) frequently co-occurred with other criteria, indicating that a high cumulative dose served as a supporting or reinforcing factor rather than an independent diagnostic standard. In cases such as P3, P55, and P61, combined with Criterion 3 or 4, this signifies a more refractory subgroup where disease progressed despite receiving high-dose RAI therapy within a relatively short period.

In summary, nearly all patients demonstrated clear evidence of disease progression despite adequate RAI therapy, characterized by persistently and significantly elevated stimulated thyroglobulin, imaging-confirmed lesion enlargement or new lesions, or clinical mortality. Based on the correspondence between inclusion criteria and clinical phenotypes, the RAIR-DTC cases in this cohort can be categorized into three main phenotypes: (i) a primary non-avid type (primarily corresponding to Criterion 1), where metastases show no iodine uptake from the initial treatment, with metastatic lesions confirmed by other imaging modalities such as ¹⁸F-FDG PET/CT; (ii) a heterogeneous or focally avid type (corresponding to Criterion 3), where patients harbor both iodine-avid and non-avid lesions, demonstrating spatial heterogeneity; and (iii) a progressing-avid type, including lesions that retain iodine uptake but progress within one year (Criterion 4), or those that exhibit a gradual loss of iodine avidity over a prolonged clinical course (Criterion 2).

**Supplementary Figure 1. Genomic alteration landscape, mutation distribution differences between recurrent and non-recurrent subgroups, and prognostic value of *TERTp* mutations in 424 DTC Patients.** **(A)** Left: Distribution of the number of alterations per patient sample (median of 1 alteration per sample). Middle: Distribution of variant types and their corresponding single nucleotide variant (SNV) classifications. Right: The top 10 most frequently mutated genes. **(B)** Comparison of mutation rates for core driver genes between the recurrent (n=80) and non-recurrent (n=308) groups. **(C)** Comparison of progression-free survival curves between DTC patients with *TERTp* mutation and those with wild-type *TERTp*.

**Supplementary Figure 2.** **Correlation of dynamic sTg changes with imaging-biomarker profiles and evolution of sTg-centered disease affinity patterns in DTC progressing to RAIR-DTC.** **(A)** Receiver operating characteristic (ROC) curve for sTg before initial RAI in predicting RAIR-DTC development, with the optimal cutoff value and area under the curve (AUC) annotated. **(B)** Scatter plot showing the association between average positive radioiodine-avid lesions and average positive FDG-avid lesions, stratified by ¹³¹I-WBS and ^18^F-FDG PET/CT status. Point color encodes sTg level in RAIR-DTC (≤50 to >500 ng/mL), and point size indicates the number of patients (5, 10, 15). **(C)** Stacked bar graph depicting the percentage of patients with ^131^I-WBS(-)_FDG(+) and ^131^I-WBS(+)_FDG(+) profiles across sTg strata (≤50, 51-100, 101-200, 201-500, >500 ng/mL) in RAIR-DTC. **(D)** Stratified scatter plots showing the relationship between positive radioiodine-avid lesions and positive FDG-avid lesions in each sTg stratum. Point color denotes imaging subgroups stratified by ^131^I-WBS and ^18^F-FDG PET/CT status. Point size reflects sTg level (larger points indicate higher sTg).

**Supplementary Figure 3. Screening flow diagram of previous research**

Enter keywords in PubMed database: (Stimulated serum thyroglobulin) AND (Differentiated thyroid cancer)

442 references identified by database search

437 references for filtering

5 duplicated

158 references excluded: 71 reviews, 5 meta-analysis, 27 case reports, 37 clinical trials, 9 non-English references and 9 other animals references

52 non-medicine references

90 full texts reviewed

38 potentially eligible studies

279 abstract screened

189 abstracts excluded: 105 with obviously irrelevant patients,treatments, biomarkers, and/or outcomes, and 84 non-related

**Supplementary Figure 4. Impact of *BRAFV600E* versus *BRAFV600E*/*TERTp* co-mutation on clinical characteristics and prognosis in RAIR-DTC.** **(A)** Comparison of age between RAIR-DTC patients with *BRAFV600E* mutation alone and those with *BRAFV600E*/*TERTp* co-mutation. **(B)** Correlation analysis between cumulative RAI dose and sTg level at RAIR-DTC diagnosis for patients with *BRAFV600E* mutation alone versus *BRAFV600E*/*TERTp* co-mutation. **(C)** Comparison of tumor size between the *BRAFV600E* and *BRAFV600E*/*TERTp* co-mutation groups in RAIR-DTC. **(D)** Comparison of cumulative RAI therapy dose between the *BRAFV600E* and *BRAFV600E*/*TERTp* co-mutation groups in RAIR-DTC patients, stratified by different tumor stages. **(E)** Comparison of sTg levels between the *BRAFV600E* and *BRAFV600E*/*TERTp* co-mutation groups in RAIR-DTC, stratified by different tumor stages. **(F)** Comparison of PFS curves between RAIR-DTC patients with *BRAFV600E* mutation alone and those with *BRAFV600E*/*TERTp* co-mutation, with the number at risk indicated at different time points.

| **Supplementary Table 1. Recurrence risk assessment of postoperative DTC** | | |
| --- | --- | --- |
| **Risk Level** | **Recurrence Probability** | **Key Features (Meets one criterion to be classified in this category)** |
| Low | <10% | Tumor ≤4 cm, single lesion, no or minimal LNM (≤5 nodes, each ≤2 mm) |
| Low-intermediate | 10-15% | Tumor with minimal ETE, unilateral multiple lesions, small amount of LNM |
| Intermediate-high | 16-30% | Bilateral multiple lesions, lateral cervical lymph node metastasis <3 cm, vascular invasion, or aggressive pathological type |
| High | >30% | Tumor with obvious ETE, lymph nodes ≥3 cm, distant metastasis, or incomplete surgical resection |

| **Supplementary Table 2. Clinical baseline characteristics of DTC** | |
| --- | --- |
| **Characteristics (N=424)** | **N (%) or median** |
| **Age at diagnosis** | 45.5±13.9 |
| Median | 44 |
| Range | 16-91 |
| < 55 years old | 307 (72.41) |
| ≥ 55 years old | 117 (27.59) |
| **Gender** | |
| Male | 129 (30.42) |
| Female | 295 (69.58) |
| **Gene mutations** | |
| SNV | 352 (83.02) |
| FA | 36 (8.49) |
| Negative | 36 (8.49) |
| **Tumor size (cm)** | 1.8±1.1 |
| **Histology** | |
| PTC | 405 (95.52) |
| cPTC | 376 (88.68) |
| PTMC | 2 (0.47) |
| tcvPTC | 4 (0.94) |
| fvPTC | 6 (1.42) |
| dsvPTC | 3 (0.71) |
| ocaPTC | 1 (0.24) |
| cPTC_fvPTC | 9 (2.12) |
| cPTC_tcvPTC | 1 (0.24) |
| cPTC_hvPTC | 1 (0.24) |
| cPTC_fvPTC_tcvPTC | 2 (0.47) |
| FTC | 14 (3.30) |
| ATC | 1 (0.24) |
| cPTC+FTC | 2 (0.47) |
| cPTC+PDTC | 2 (0.47) |
| **Initial sTg (median, ng/mL)** | 4.8 |
| **Multifocality** | |
| Singleness | 209 (49.29) |
| Multifocality | 215 (50.71) |
| **ETE** | 150 (35.38) |
| **Vascular invasion** | 67 (15.80) |
| **LNM** | 327 (77.12) |
| **Stage** | |
| I | 316 (74.53) |
| II | 33 (7.78) |
| III | 29 (6.84) |
| IVa | 15 (3.54) |
| IVb | 19 (4.48) |
| IVc | 12 (2.83) |
| **AJCC stage** | |
| T stage | |
| T1 | 217 (51.18) |
| T2 | 50 (11.79) |
| T3 | 116 (27.36) |
| T4 | 41 (9.67) |
| N stage | |
| N0 | 84 (19.81) |
| N1a | 122 (28.77) |
| N1b | 200 (47.17) |
| Nx | 18 (4.25) |
| M stage | |
| M0 | 377 (88.92) |
| M1 | 47 (11.08) |
| **Cumulative RAI dose (mCi)** | |
| < 600 | 416 (98.11) |
| ≥ 600 | 8 (1.88) |
| **Recurrence** | |
| Y | 85 (20.05) |
| N | 339 (79.95) |
| **Therapeutic times of RAI** | |
| < 3 | 407 (95.99) |
| ≥ 3 | 17 (4.01) |
| **RAIR-DTC** | |
| Y | 360 (84.91) |
| N | 64 (15.09) |

| **Supplementary Table.3 Inclusion criteria of RAIR-DTC** | |
| --- | --- |
| **Diagnostic criteria of RAIR-DTC** | **Number of patients** |
| 1. Metastases that are RAI-nonavid on the initial posttreatment ^131^I-WBS | 31 |
| 2. Functional metastases that originally are RAI-avid gradually lose their iodine uptake ability | 1 |
| 3. Some metastases are RAI-avid, while others are RAI-nonavid, which can be detected by other imaging methods such as ^18^F-FDG PET/CT, CT, or MRI | 8 |
| 4. RAI-avid metastases maintain the iodine uptake ability, but the disease progress within one year after RAI therapy, including gradual growth of the lesions, the appearance of new lesions, or a continuous increase of Tg or Tg-antibody (TgAb) levels | 27 |
| 5. Cumulative RAI dose >600 mCi | 6 |

| **Supplementary Table 4. Clinical course in 64 patients from initial RAI to RAIR-DTC** | | |
| --- | --- | --- |
| **Patients** | **Inclusion criteria** | **Detailed description of diagnostic basis** |
| P1 | 1 | ^131^I-WBS indicates non-iodine uptake. The metastatic lesions do not exhibit iodine uptake in the ^131^I-WBS after the initial treatment, but the disease progresses. |
| P2 | 1 | ^131^I-WBS indicates non-iodine uptake. The metastatic lesions do not exhibit iodine uptake in the ^131^I-WBS after the initial treatment, but the disease progresses. |
| P3 | 3, 5 | The ^131^I-WBS showed positive uptake of iodine in the neck, but the ^18^F-FDG PET/CT scan indicated positive metastases in the lungs and bones (not taking up iodine but detected), and the cumulative dose of ^131^I was 900 mCi > 600 mCi. |
| P4 | 4 | The ^131^I-WBS shows bilateral lung iodine uptake, with the sTg level rising from 48.1 to 1412.1, and the disease progression. |
| P5 | 4 | The ^131^I-WBS test was positive (indicating iodine uptake), but the sTg level increased from 52.3 to 601, and the disease progressed. |
| P6 | 1 | ^131^I-WBS indicates non-iodine uptake. The metastatic lesions do not exhibit iodine uptake in the ^131^I-WBS after the initial treatment, but the disease progresses. |
| P7 | 1 | ^131^I-WBS indicates non-iodine uptake. The metastatic lesions do not exhibit iodine uptake in the ^131^I-WBS after the initial treatment, but the disease progresses. |
| P8 | 1 | ^131^I-WBS indicates non-iodine uptake. The metastatic lesions do not exhibit iodine uptake in the ^131^I-WBS after the initial treatment, but the disease progresses. |
| P9 | 4 | The ^131^I-WBS shows multiple bone iodine uptake, and the sTg level increased from 201 to 528, indicating disease progression. |
| P10 | 1 | ^131^I-WBS indicates non-iodine uptake. The metastatic lesions do not exhibit iodine uptake in the ^131^I-WBS after the initial treatment, but the disease progresses. |
| P11 | 1 | ^131^I-WBS indicates non-iodine uptake. The metastatic lesions do not exhibit iodine uptake in the ^131^I-WBS after the initial treatment, but the disease progresses. |
| P12 | 1 | The ^131^I-WBS test showed no iodine uptake, but the sTg level increased from 9.4 to 755.4, and the disease progressed. |
| P13 | 1 | ^131^I-WBS indicates non-iodine uptake. The metastatic lesions do not exhibit iodine uptake in the ^131^I-WBS after the initial treatment, but the disease progresses. |
| P14 | 1 | ^131^I-WBS indicates non-iodine uptake. The metastatic lesions do not exhibit iodine uptake in the ^131^I-WBS after the initial treatment, but the disease progresses. |
| P15 | 3 | The ^131^I-WBS shows iodine uptake in the cervical lymph nodes, but no iodine uptake in the lungs, and the ^18^F-FDG PET/CT showed positive results in both the neck and the lungs. |
| P16 | 1 | ^131^I-WBS indicates non-iodine uptake. The metastatic lesions do not exhibit iodine uptake in the ^131^I-WBS after the initial treatment, but the disease progresses. |
| P17 | 3 | The ^131^I-WBS showed iodine uptake in the clavicular region and parasternal lymph nodes, while the ^18^F-FDG PET/CT revealed positive results in bilateral lungs. |
| P18 | 3 | The ^131^I-WBS showed iodine uptake in the multiple bones, but not in the bilateral lungs. |
| P19 | 1 | ^131^I-WBS indicates non-iodine uptake. The metastatic lesions do not exhibit iodine uptake in the ^131^I-WBS after the initial treatment, but the disease progresses. |
| P20 | 2 | The ^131^I-WBS shows radioactive iodine uptake, but the period from the initial treatment to the diagnosis of RAIR-DTC was very long (from August 1992 to February 2023), suggesting a gradual loss of iodine uptake ability. |
| P21 | 1 | ^131^I-WBS indicates non-iodine uptake. The metastatic lesions do not exhibit iodine uptake in the ^131^I-WBS after the initial treatment, but the disease progresses. |
| P22 | 1 | ^131^I-WBS indicates non-iodine uptake. The metastatic lesions do not exhibit iodine uptake in the ^131^I-WBS after the initial treatment, but the disease progresses. |
| P23 | 1 | ^131^I-WBS indicates non-iodine uptake. The metastatic lesions do not exhibit iodine uptake in the ^131^I-WBS after the initial treatment, but the disease progresses. |
| P24 | 1 | ^131^I-WBS indicates non-iodine uptake. The metastatic lesions do not exhibit iodine uptake in the ^131^I-WBS after the initial treatment, but the disease progresses. |
| P25 | 4 | The ^131^I-WBS test shows positive for bone (iodine uptake) and an extremely high sTg level (74,568), indicating disease progression. |
| P26 | 1 | The ^131^I-WBS test showed no iodine uptake, but the ^18^F-FDG PET/CT test was positive, and the sTg level increased from 464 to 4464, indicating disease progression. |
| P27 | 1 | The ^131^I-WBS showed no iodine uptake, but the sTg level increased from 20.7 to 705.4, and the patient was dead. |
| P28 | 1 | ^131^I-WBS indicates non-iodine uptake. The metastatic lesions do not exhibit iodine uptake in the ^131^I-WBS after the initial treatment, but the disease progresses. |
| P29 | 4 | The ^131^I-WBS shows radioactive iodine uptake in the bilateral lungs and bones,and the sTg level increased from 46.6 to 704, indicating disease progression. |
| P30 | 1 | ^131^I-WBS indicates non-iodine uptake. The metastatic lesions do not exhibit iodine uptake in the ^131^I-WBS after the initial treatment, but the disease progresses. |
| P31 | 4 | The ^131^I-WBS showed no iodine uptake, but the sTg level increased from 500 to 2595, and the disease progressed. |
| P32 | 1, 4 | The ^131^I-WBS was negative, and the ssTg level increased from 26.7 to 289. The survival status was death, suggesting disease progression. |
| P33 | 1 | ^131^I-WBS indicates non-iodine uptake. The metastatic lesions do not exhibit iodine uptake in the ^131^I-WBS after the initial treatment, but the disease progresses. |
| P34 | 4 | The ^131^I-WBS shows iodine uptake in bilateral lungs and bones, and the sTg level increased from 57.7 to 1038, reflecting the progression of the disease. |
| P35 | 4 | The ^131^I-WBS showed iodine uptake in the thoracic vertebrae and the spinal cord, and the sTg level increased from 12.1 to 4997, indicating disease progression. |
| P36 | 1 | ^131^I-WBS indicates non-iodine uptake. The metastatic lesions do not exhibit iodine uptake in the ^131^I-WBS after the initial treatment, but the disease progresses. |
| P37 | 1 | ^131^I-WBS indicates non-iodine uptake. The metastatic lesions do not exhibit iodine uptake in the ^131^I-WBS after the initial treatment, but the disease progresses. |
| P38 | 1 | ^131^I-WBS indicates non-iodine uptake. The metastatic lesions do not exhibit iodine uptake in the ^131^I-WBS after the initial treatment, but the disease progresses. |
| P39 | 1 | ^131^I-WBS indicates non-iodine uptake. The metastatic lesions do not exhibit iodine uptake in the ^131^I-WBS after the initial treatment, but the disease progresses. |
| P40 | 3, 4 | The ^131^I-WBS showed radioactive iodine uptake in the neck, and ^18^F-FDG PET/CT showed positive in the lungs and bones. The sTg level increased from 47.4 to 743.3, suggesting disease progression. |
| P41 | 1 | ^131^I-WBS indicates non-iodine uptake. The metastatic lesions do not exhibit iodine uptake in the ^131^I-WBS after the initial treatment, but the disease progresses. |
| P42 | 1 | ^131^I-WBS indicates non-iodine uptake. The metastatic lesions do not exhibit iodine uptake in the ^131^I-WBS after the initial treatment, but the disease progresses. |
| P43 | 3 | The ^131^I-WBS showed radioactive iodine uptake in the subclavian and mediastinal lymph nodes, and ^18^F-FDG PET/CT showed positive. The sTg level increased from 15.1 to 399.2, suggesting disease progression. |
| P44 | 3, 4 | The ^131^I-WBS showed radioactive iodine uptake in the bilateral cervical lymph nodes, and ^18^F-FDG PET/CT showed positive in the bilateral lungs and bones. The sTg level increased from 4.8 to 425.0, suggesting disease progression. |
| P45 | 1 | ^131^I-WBS indicates non-iodine uptake. The metastatic lesions do not exhibit iodine uptake in the ^131^I-WBS after the initial treatment, but the disease progresses. |
| P46 | 1 | ^131^I-WBS indicates non-iodine uptake. The metastatic lesions do not exhibit iodine uptake in the ^131^I-WBS after the initial treatment, but the disease progresses. |
| P47 | 3, 4 | The ^131^I-WBS showed radioactive iodine uptake in the right supraclavicular and hilar lymph nodes, and ^18^F-FDG PET/CT showed positive in the bilateral lungs and sternum. The sTg level increased from 58.8 to 848.4, suggesting disease progression. |
| P48 | 1 | ^131^I-WBS indicates non-iodine uptake. The metastatic lesions do not exhibit iodine uptake in the ^131^I-WBS after the initial treatment, but the disease progresses. |
| P49 | 1 | ^131^I-WBS indicates non-iodine uptake. The metastatic lesions do not exhibit iodine uptake in the ^131^I-WBS after the initial treatment, but the disease progresses. |
| P50 | 1 | ^131^I-WBS indicates non-iodine uptake. The metastatic lesions do not exhibit iodine uptake in the ^131^I-WBS after the initial treatment, but the disease progresses. |
| P51 | 1 | The ^131^I-WBS test showed no iodine uptake, although the sTg level was less than 0.04 because sTg is suppressed by TSH. |
| P52 | 1 | The ^131^I-WBS shows no iodine uptake, but the ^18^F-FDG PET/CT indicates positive results for hilar lymph nodes and multiple bones. |
| P53 | 4 | The ^131^I-WBS showed iodine uptake in the neck, clavicle, mediastinal lymph nodes, and the sTg level ranged from 6.9 to 458.1, indicating disease progression. |
| P54 | 4 | The ^131^I-WBS showed radioactive iodine uptake in the bilateral lungs and multiple bones. The sTg level increased from 11.2 to 864.4. |
| P55 | 4, 5 | The ^131^I-WBS showed radioactive iodine uptake in the multiple bones. The sTg level increased from 16.0 to 875.9, and the time from the initial treatment to the diagnosis of RAIR-DTC was short (from September 2013 to May 2022), suggesting disease progression. Moreover, the cumulative dose reached 1100 mCi. |
| P56 | 4 | The ^131^I-WBS shows iodine uptake in the cervical lymph nodes, lungs and bones, |
| P57 | 1 | ^131^I-WBS indicates non-iodine uptake. The metastatic lesions do not exhibit iodine uptake in the ^131^I-WBS after the initial treatment, but the disease progresses. |
| P58 | 1 | ^131^I-WBS indicates non-iodine uptake. The metastatic lesions do not exhibit iodine uptake in the ^131^I-WBS after the initial treatment, but the disease progresses. |
| P59 | 4 | The ^131^I-WBS shows iodine uptake in the bilateral lungs and bone, and the sTg level increased from 1.2 to 633.8, indicating disease progression. |
| P60 | 4 | The ^131^I-WBS showed no iodine uptake, but the sTg level increased from 0.5 to 900.3, and the patient was dead. |
| P61 | 4, 5 | The ^131^I-WBS showed radioactive iodine uptake in the multiple bones. The sTg level increased from 62.31 to 438.5, and the time from the initial treatment to the diagnosis of RAIR-DTC was short (from September 2019 to December 2019). Moreover, the cumulative dose reached 600 mCi. |
| P62 | 4 | The ^131^I-WBS shows iodine uptake in bilateral lungs, and the sTg level increased from 60.0 to 811.0, reflecting the progression of the disease. |
| P63 | 4 | The ^131^I-WBS shows iodine uptake in multiple bones, and the sTg level increased from 0.3 to 302.6, reflecting the progression of the disease. |
| P64 | 4 | The ^131^I-WBS shows bilateral lung and multiple bone iodine uptake, and the sTg level increased from 39.0 to 189.9. |

| **Supplementary Table 5. 38 previous literatures related to predictive cut-off value of sTg** | | | | |
| --- | --- | --- | --- | --- |
| **No.** | **Reference** | **Patients** | **sTg cut-off** | **Clinical significance** |
| This study | TERT Promoter Mutations Drive Targeted Therapy Resistance and Poor Prognosis in Radioactive Iodine-Refractory Differentiated Thyroid Cancer: A Clinical Study Based on a Multimodal Machine Learning Model for Predicting RAIR-DTC | RAIR-DTC and non-RAIR-DTC | 10.77 ng/mL | 1. Systematically define the genomic and clinicopathological features distinguishing RAIR-DTC from RAI-avid patients;  2. Decipher the sTg-driven evolution of metastatic affinity patterns and dissemination dynamics in RAIR-DTC;  3. Elucidate how specific genetic alterations dictate treatment sequence, metabolic phenotype, and response to targeted drugs;  4. Develop and validate a machine learning (LASSO)-derived prognostic model integrating key molecular and clinical variables for precise, individualized risk stratification of RAIR-DTC. |
| 1 | Prognostic value of postoperative stimulated thyroglobulin in differentiated thyroid cancer | DTC without distant metastasis | 3.15 ng/mL | Evaluate the prognostic value of postoperative Tg in DTC for subsequent disease-free status. |
| 2 | Prognostic value of pre‐ablation stimulated thyroglobulin in children and adolescents with differentiated thyroid cancer | DTC in children and adolescents | 17.8 ng/mL | Investigate the prognostic value of pre-ablation stimulated thyroglobulin (s-Tg) in children and adolescents with differentiated thyroid cancer |
| 3 | Combination of Stimulated Thyroglobulin and Antithyroglobulin Antibody Predicts the Efficacy and Prognosis of (131)I Therapy in Patients With Differentiated Thyroid Cancer Following Total Thyroidectomy | DTC | 2.99 ng/mL | Independent risk factors for unsuccessful ablation |
| 4 | Only peak thyroglobulin concentration on day 1 and 3 of rhTSH-aided RAI adjuvant treatment has prognostic implications in differentiated thyroid cancer | DTC（rhTSH stimulation） | Tg1: 0.7 ng/mL; Tg3: 1.4 ng/mL; Note: Tg was measured on day 1 (Tg1; at the time of the first rhTSH injection), day 3 (Tg3; 1 day after the second, final rhTSH injection) | Predict structural recurrence |
| 5 | A novel nomogram integrated with preablation stimulated thyroglobulin and thyroglobulin/thyroid-stimulating hormone ratio to predict the therapeutic response of intermediate‑ and high‑risk differentiated thyroid cancer patients | Intermediate and high-risk DTC | 4.7 | Predict the therapeutic response of intermediate and high risk DTC |
| 6 | Stimulated serum thyroglobulin but not RAIU level is a prognostic factor for ablation efficacy with a 3.7GBq (100mCi) fixed (131)I dose in patients with differentiated thyroid cancer | DTC without distant metastasis | sTg < 2, 2-5, 5-10, ≥10 ng/mL | Determine prognostic value of radioactive iodine uptake (RAIU) and stimulated thyroglobulin (sTg) regarding the ablation efficacy in patients with differentiated thyroid cancer |
| 7 | Undetectable thyroglobulin makes (123)I whole-body scan and stimulated thyroglobulin obsolete in follow-up care of differentiated thyroid cancer | DTC | 1 ng/mL | Determine the prognostic utility of high-sensitive Tg and the need for other diagnostic tests in DTC |
| 8 | Prognostic value of preablative stimulated thyroglobulin and its decline after (131)I therapy in differentiated thyroid cancer | DTC without distant metastasis | Pre-131I: 14.55 μg/L | Assess the prognostic value of stimulated thyroglobulin (sTg) levels before and after thefirst131I treatment in DTc patients without distant metastases |
| 9 | BRAF V600E Status and Stimulated Thyroglobulin at Ablation Time Increase Prognostic Value of American Thyroid Association Classification Systems for Persistent Disease in Differentiated Thyroid Cancer | DTC with recurrence risk of structural disease | 8.9 ng/mL | Evaluate the prognostic role of A-hTg combined with the BRAFV600E status in association with the revised American Thyroid Association (ATA) risk stratification |
| 10 | Role of Postoperative Stimulated Thyroglobulin as Prognostic Factor for Differentiated Thyroid Cancer in Children and Adolescents | DTC in children and adolescents | 31.5 ng/mL | Evaluate potential prognostic factors in children and adolescents with DTC, with special emphasis on sPOTg analysis. |
| 11 | Prognostic Value of Stimulated Thyroglobulin Levels at the Time of Radioiodine Administration in Differentiated Thyroid Cancer | DTC | <10 µg/L | Assess the prognostic value of stimulated thyroglobulin (sTg) and Tg-related parameters prior to and immediately after radioactive iodine (RAI) administration |
| 12 | Early preablation rhTSH-stimulated thyroglobulin predicts outcome of differentiated thyroid cancer (DTC) patients | Low and intermediate-risk DTC (rhTSH) | Basal Tg: 1.55 ng/mL; Early stimulation of Tg: 2.6 ng/mL; Late stage: 4.9 ng/mL | Evaluate the predictive role of preablation-Tg in patients underwent RaIT after rhTSH stimulation |
| 13 | Genetic mutations and prognostic indicators in differentiated thyroid cancer: a molecular perspective | Intermediate and high-risk DTC | 49.2 ng/mL | Examine the relationship between the presence of metastatic disease and Tg |
| 14 | Prognostic factors in children and adolescents with differentiated thyroid carcinoma treated with total thyroidectomy and RAI: a real-life multicentric study | DTC in children and adolescents | 27.2 ng/mL | Investigate the main prognostic factors associated with treatment response at 1 year after radioactive iodine therapy (RAIT) and the last disease status in pediatric patients affected by differentiated thyroid carcinoma (DTC). |
| 15 | The first postoperative-stimulated serum thyroglobulin is a prognostic factor for thyroid microcarcinomas | DTC(PMTC) | 1.6 ng/mL | Assess whether the first postoperative stimulated thyroglobulin measurement is a prognostic factor in patients with microcarcinoma |
| 16 | Prognostic factors in children and adolescents with differentiated thyroid cancer treated with total thyroidectomy and radioiodine therapy: a retrospective two-center study from China | DTC in children and adolescents | 20 ng/mL | Explore the main prognostic factors affecting the final disease status in children and adolescents with differentiated thyroid cancer (caDTC) following total thyroidectomy and radioiodine therapy (RAIT). |
| 17 | Value of stimulated pre-ablation thyroglobulin as a prognostic marker in patients with differentiated thyroid carcinoma treated with radioiodine | DTC | 4.4 ng/mL | Determine which Thyroglobulin (Tg) level after levothyroxine (LT 4) withdrawal (stimulated thyroglobulin - sTg) measured before radioiodine therapy (RAIT) is able to predict incomplete response to treatment of differentiated thyroid carcinoma (DTC) with greater sensitivity and specificity one year after initial treatment with I 131. |
| 18 | Prognostic Implications of Preablation Stimulated Tg: A Retrospective Analysis of 2500 Thyroid Cancer Patients | DTC | ≤10.1 ng/mL | Examine the association between pre-ablation stimulated thyroglobulin (ps-Tg) and persistent and recurrent disease in DTC patients and investigate whether incorporation of ps-Tg could provide a more individualized estimate of clinical outcomes. |
| 19 | Thyroglobulin measurement is the most powerful outcome predictor in differentiated thyroid cancer: a decision tree analysis in a European multicenter series | DTC | Structural disease: 63.1 ng/mL; Biochemical diseases: 41.2 ng/mL; Low risk: 8.9 ng/mL | Develop a predictive model for recurrent disease in DTC patients treated by surgery and 131I by adopting a decision tree model. |
| 20 | Prognostic implications of radioiodine avidity and serum thyroglobulin in differentiated thyroid carcinoma with distant metastasis | DTC with distant metastasis | sTg ≤ 215 ng/mL | Evaluate factors that affect the survival of patients with DTC who present with distant metastasis. |
| 21 | Thyroglobulin as early prognostic marker to predict remission at 18-24 months in differentiated thyroid carcinoma | DTC | <8.55 μg/L | Evaluate the role of baseline-stimulated Tg as early prognostic marker of DTC. |
| 22 | Preablative Stimulated Thyroglobulin and Thyroglobulin Reduction Index as Decision-Making Markers for Second Radioactive Iodine Therapy in Patients with Structural Incomplete Response | DTC with incomplete structural response | presTg1 < 6.6 ng/mL; presTg2 < 1.2 ng/mL; TRI > 74.2% | Evaluate the value of preablative stimulated thyroglobulin (presTg) and thyroglobulin reduction index (TRI) to predict the different responses to second radioactive iodine (RAI) therapy in differentiated thyroid cancer (DTC) patients with structural incomplete response (SIR). |
| 23 | Recombinant human TSH stimulated thyroglobulin levels at remnant ablation predict structural incomplete response to treatment in patients with differentiated thyroid cancer | DTC(rhTSH stimulation) | RAI: 4.64 ng/mL; THW-sTg: 2.41 ng/mL; rhTSH-sTg: 1.02 ng/mL | Evaluate the prognostic impacts and best cutoff values of sTg levels under recombinant human thyroid stimulating hormone (rhTSH) treatment at RA and at follow-up. |
| 24 | Response to Therapy Assessment in Intermediate-Risk Thyroid Cancer Patients: Is Thyroglobulin Stimulation Required? | Intermediate risk DTC | 1 ng/mL or 0.2 ng/mL | Explore whether it is necessary to conduct Tg testing under rhTSH stimulation when evaluating the initial treatment response in patients with intermediate-risk differentiated thyroid cancer. |
| 25 | The Use of Post-ablation Stimulated Thyroglobulin in Predicting Clinical Outcomes in Differentiated Thyroid Carcinoma - What Cut-off Values Should We Use? | DTC | 1.0 ng/mL | Provide data on the prognostic value of sTg at different levels of sensitivities and specificities. |
| 26 | Serum thyroglobulin concentrations predict disease-free remission and death in differentiated thyroid carcinoma | DTC | Pre-ablation: 27.5 μg/L; After 6 months: 10 μg/L | Investigate the prognostic values of serum Tg for disease-free remission and death, measured at fixed time-points after initial therapy using receiver operator characteristic (ROC) curve analyses. |
| 27 | Prognostic implication of thyroglobulin and quantified whole body scan after initial radioiodine therapy on early prediction of ablation and clinical response for the patients with differentiated thyroid cancer | DTC | <1 ng/mL 1-5 ng/mL >5 ng/mL | Investigate predictors for successful ablation and disease-free status after high-dose radioiodine therapy in patients with DTC |
| 28 | Predictive Role of Serum Thyroglobulin after Surgery and before Radioactive Iodine Therapy in Patients with Thyroid Carcinoma | PTC | Predict nodal disease: Tg-30 1.30 ng/mL; Tg0 5.70 ng/mL; Tg+7 78.23 ng/mL Predict distal disease: Tg-30 16 ng/mL; Tg0 32.98 ng/mL; Tg+7 100.07 ng/mL | The first Tg measurement is highly indicative and predictive of the future development of metastases |
| 29 | Recombinant human thyrotropin-stimulated thyroglobulin level at the time of radioactive iodine ablation is an independent prognostic marker of differentiated thyroid carcinoma in the setting of prophylactic central neck dissection | DTC | 1.79 ng/mL | Investigate the value of the stimulated thyroglobulin (Tg) level at the time of recombinant human thyrotropin (rhTSH)-aided remnant ablation for predicting disease status 1 year later in patients with DTC who underwent total thyroidectomy with central neck dissection (CND). |
| 30 | Thyroglobulin levels measured at the time of remnant ablation to predict response to treatment in differentiated thyroid cancer after thyroid hormone withdrawal or recombinant human TSH | DTC(rhTSH or THW) | rhTSH: 8 ng/mL; THW: 22 ng/mL; Low-risk THW: 12 ng/mL; Intermediate-high risk THW: 16 ng/mL | Evaluate the prognostic value of stimulated thyroglobulin levels at the moment of remnant ablation for predicting an initial excellent or a structural incomplete response to treatment according to the risk of recurrence in patients with differentiated thyroid cancer. |
| 31 | Predictive value for disease progression of serum thyroglobulin levels measured in the postoperative period and after (131)I ablation therapy in patients with differentiated thyroid cancer | DTC without distant metastasis | Tg1 > 30 ng/mL; Tg2 > 10 ng/mL | Evaluate and compare in thyroid cancer patients the predictive value for disease progression of thyroglobulin (Tg) levels measured under thyroid-stimulating hormone (TSH) stimulation, in the postoperative period just before (131)I ablative therapy and at the time of control 6-12 mo later. |
| 32 | Prognostic value of postsurgical stimulated thyroglobulin levels after initial radioactive iodine therapy in well-differentiated thyroid carcinoma | DTC | 30.0 μg/L (IQR 13.6, 55.0 μg/L); 5 μg/L (IQR 3.0, 9.98 μg/L) | Explore predictors of future positivity of stimulated thyroglobulin (>2 μg/L) after initial radioactive iodine treatment |
| 33 | Optimization of serum thyroglobulin measured at different time points for prognostic evaluation in differentiated thyroid carcinoma patients | DTC (rhTSH) | Non-AR: D-7Tg-0.6 ng/mL; Non-AR: D0Tg-2.6 ng/mL; Non-AR: D2Tg-3.7 ng/mL; PRD: D-7Tg-0.8 ng/mL; PRD: D0Tg-4.0 ng/mL; PRD: D2Tg-6.0 ng/mL | Investigate serum Tg prognostic values measured at different time points before and after radioactive iodine (RAI) therapy prepared with recombinant human TSH (rhTSH) in DTC patients |
| 34 | Prognostic significance of FDG PET/CT on the follow-up of patients of differentiated thyroid carcinoma with negative 131I whole-body scan and elevated thyroglobulin levels | DTC (elevated Tg and negative ^131^I-WBS) | TSH suppression: 1.9 ng/mL; TSH stimulation: 38.2 ng/mL | 1. Determine the clinical and histopathologic factors that are related to FDG avidity in the recurrence/metastases of patients with differentiated thyroid carcinoma (DTC) who present with elevated thyroglobulin (Tg) levels and negative 131I whole-body scans (WBSs). 2. Clarify Tg cutoff levels in this setting. 3. Evaluate the impact of PET/CT on patient management strategies and hence to critically look into the importance of PET/CT in combination with Tg in clinical decision making. |
| 35 | Comparison of models predicting efficacy of radioiodine therapy in patients with differentiated thyroid cancer | DTC | 7.22 ng/mL | Assess predictive models for RAI therapy efficacy in patients with DTC and to identify thyroid biomarker cutoff values. |
| 36 | Combination of initial stimulation thyroglobulins and staging system by revised ATA guidelines can elaborately discriminate prognosis of patients with differentiated thyroid carcinoma after high-dose remnant ablation | PTC | 5.22 ng/mL | Evaluate prognostic role of thyroglobulin (Tg) levels at the time of ablation (A-Tg) and stimulation Tg levels at 6-12 months after remnant ablation (S-Tg) combined with revised American Thyroid Association (ATA) guidelines risk stratification. |
| 37 | A new functional parameter measured at the time of ablation that can be used to predict differentiated thyroid cancer recurrence during follow-up | DTC | Tg/(TSH×24-h I-131 uptake) | Addresses the questions whether patients with a high risk for recurrent thyroid cancer can be identified at initial stage, i.e. at the time of ablation. |
| 38 | Stimulated thyroglobulin at recombinant human TSH-aided ablation predicts disease-free status one year later | DTC without distant metastasis | 7.2 ng/mL | Determine whether stimulated Tg levels, measured at the time of remnant ablation performed under recombinant human TSH (rhTSH) stimulation, has value in predicting absence of detectable disease 1 year after radioiodine therapy and to compare the results obtained with this approach with a cohort of patients submitted to ablation after THW. |

| **Supplementary Table 6. Comparison of clinical characteristics between *TERTp wild type* and *TERTp mutant patients*** | | | |
| --- | --- | --- | --- |
| **Characteistic** | ***TERTp* wild type (n=36)** | ***TERTp* mutant (n=28)** | ***P* value** |
| **Age at diagnosis** | | | |
| Median | 64 | 65 | ns |
| Range | 34-80 | 47-78 |  |
| **Gender** | | | |
| Male | 11 (30.56) | 11 (39.29) | ns |
| Female | 25 (69.44) | 17 (60.71) |  |
| **Histology** | | | |
| PTC | 29 (80.56) | 25 (89.29) | ns |
| cPTC | 28 (77.78) | 24 (85.71) |  |
| PTMC | 1 (2.78) | 1 (3.57) |  |
| FTC | 6 (16.67) | 3 (10.71) |  |
| ATC | 1 (2.78) | 0 (0.00) |  |
| **Multifocality** | | | |
| Singleness | 26 (72.22) | 15 (53.57) | ns |
| Multifocality | 10 (27.78) | 13 (46.43) |  |
| **ETE** | 23 (63.89) | 20 (71.43) | ns |
| **Vascular invasion** | 17 (47.22) | 19 (67.86) | ns |
| **LNM** | 15 (41.67) | 19 (67.86) | **<0.05** |
| **Stage** | | | |
| I-II | 16 (44.44) | 8 (28.57) | ns |
| III-IV | 20 (55.56) | 20 (71.43) |  |
| **TNM stage** | | | |
| T stage | | | |
| T1 | 13 (36.11) | 9 (32.14) | **<0.001** |
| T2 | 4 (11.11) | 1 (3.57) |  |
| T3 | 7 (19.44) | 5 (17.86) |  |
| T4 | 6 (16.67) | 17 (60.71) |  |
| Tx | 6 (16.67) | 3 (10.71) |  |
| N stage | | | |
| N0 | 15 (41.67) | 7 (25.00) | **<0.05** |
| N1a | 2 (5.56) | 3 (10.71) |  |
| N1b | 13 (36.11) | 16 (57.14) |  |
| Nx | 6 (16.67) | 2 (7.14) |  |
| M stage | | | |
| M0 | 21 (58.33) | 15 (53.57) | ns |
| M1 | 15 (41.67) | 13 (46.43) |  |
| **MOM at RAIR_DTC** | | | |
| SOM | 24 (66.67) | 5 (17.86) | **<0.001** |
| MOM | 12 (33.33) | 23 (82.14) |  |
| **Cumulative RAI dose (mCi)** | | | |
| < 600 | 36 (100.00) | 23 (82.14) | ns |
| ≥ 600 | 0 (0.00) | 5 (17.86) |  |
| **Recurrence** | 22 (77.78) | 28 (100.00) | **<0.001** |
| **Therapeutic times of RAI** | | | |
| < 3 | 30 (83.33) | 22 (78.57) | ns |
| ≥ 3 | 6 (16.67) | 6 (21.43) |  |

| **Supplementary Table 7. Comparison of clinical characteristics between *BRAFV600E* and *BRAFV600E*/*TERTp*** | | | |
| --- | --- | --- | --- |
| **Characteistic** | ***BRAFV600E* (n=29)** | ***BRAFV600E/TERTp* (n=23)** | ***P* value** |
| **Age at diagnosis** | | | |
| Median | 64 | 66 | **<0.0001** |
| Range | 42-80 | 47-75 |  |
| **Gender** | | | |
| Male | 9 (31.03) | 10 (43.48) | ns |
| Female | 20 (68.97) | 13 (56.52) |  |
| **Histology** | | | |
| PTC | 23 (79.31) | 20 (86.96) | ns |
| cPTC | 22 (75.86) | 19 (82.61) |  |
| PTMC | 1 (3.45) | 1 (4.35) |  |
| FTC | 5 (17.24) | 3 (13.04) | ns |
| ATC | 1 (3.45) | 0 (0.00) | ns |
| **Multifocality** | | | |
| Singleness | 21 (72.41) | 12 (52.17) | ns |
| Multifocality | 8 (27.59) | 11 (47.83) |  |
| **ETE** | 19 (65.52) | 15 (65.22) | ns |
| **Vascular invasion** | 15 (51.72) | 16 (69.57) | ns |
| **LNM** | 13 (44.83) | 17 (73.91) | ns |
| **Stage** | | | |
| I | 10 (34.48) | 8 (34.78) | ns |
| II | 2 (6.90) | 0 (0.00) |  |
| III | 3 (10.34) | 1 (4.35) |  |
| IVa | 7 (24.14) | 7 (30.43) |  |
| IVb | 4 (13.79) | 2 (8.70) |  |
| IVc | 3 (10.34) | 5 (21.74) |  |
| **TNM stage** | | | |
| T stage | | | |
| T1 | 10 (34.48) | 7 (30.43) | ns |
| T2 | 2 (6.90) | 0 (0.00) |  |
| T3 | 7 (24.14) | 4 (17.39) |  |
| T4 | 6 (20.69) | 9 (39.13) |  |
| Tx | 4 (13.79) | 3 (13.04) |  |
| N stage | | | |
| N0 | 11 (37.93) | 4 (17.39) | **0.011** |
| N1a | 2 (6.90) | 3 (13.04) |  |
| N1b | 10 (34.48) | 15 (65.22) |  |
| Nx | 6 (20.69) | 1 (4.35) |  |
| M stage | | | |
| M0 | 19 (65.52) | 14 (60.87) | ns |
| M1 | 10 (34.48) | 9 (39.13) |  |
| **MOM at RAIR_DTC** | | | |
| SOM | 12 (41.38) | 5 (21.74) | **<0.001** |
| MOM | 17 (58.62) | 18 (78.26) |  |
| **Cumulative RAI dose (mCi)** | | | |
| < 600 | 29 (100.00) | 18 (78.26) | **<0.01** |
| ≥ 600 | 0 (0.00) | 5 (21.74) |  |
| **Recurrence** | 22 (75.9) | 23 (100.00) | **0.012** |
| **Therapeutic times of RAI** | | | |
| < 3 | 26 (89.66) | 18 (78.26) | ns |
| ≥ 3 | 3 (10.34) | 5 (21.74) |  |

| **Abbreviations list** | |
| --- | --- |
| RAIR-DTC | Radioactive Iodine-Refractory Differentiated Thyroid Cancer |
| DTC | Differentiated Thyroid Carcinoma |
| TERTp | TERT promoter |
| NGS | Next-generation sequencing |
| sTg | stimulated thyroglobulin |
| TSH | Thyroid-Stimulating Hormone |
| TgAb | Anti-thyroglobulin antibodies |
| RAI | Radioactive Iodine |
| WBS | Whole-Body Scan |
| FDG | Fluorodeoxyglucose |
| PET/CT | Positron Emission Tomography / Computed Tomography |
| SPECT/CT | Single-Photon Emission Computed Tomography / Computed Tomography |
| SUVmax | Maximum Standardized Uptake Value |
| NIS | Sodium-Iodide Symporter |
| MAPK | Mitogen-Activated Protein Kinase |
| OSEM | Ordered-Subsets Expectation Maximization |
| MPR | Multi-Planar Reconstruction |
| ROI | Region of Interest |
| NGS | Next-Generation Sequencing |
| FFPE | Formalin-Fixed, Paraffin-Embedded |
| PFS | Progression-Free Survival |
| AJCC | American Joint Committee on Cancer |
| ETE | Extrathyroidal Extension |
| LNM | Lymph Node Metastasis |
| FTC | Follicular Thyroid Carcinoma |
| ATC | Anaplastic Thyroid Carcinoma |
| PTC | Papillary Thyroid Carcinoma |
| cPTC | Classic PTC |
| PTMC | Papillary Thyroid Microcarcinoma |
| tcvPTC | Tall Cell Variant PTC |
| fvPTC | Follicular Variant PTC |
| dsvPTC | Diffuse Sclerosing Variant PTC |
| ocaPTC | Oncocytic Adenoma PTC |
| SNV | Single Nucleotide Variant |
| FA | Fusion Alteration |
| TKI | Tyrosine Kinase Inhibitor |
| ORR | Objective Response Rate |
| DCR | Disease Control Rate |
| LASSO | Least Absolute Shrinkage and Selection Operator |
| ROC | Receiver Operating Characteristic |
| AUC | Area Under the Curve |
| DCA | Decision Curve Analysis |
